# The value of the MIND diet in the primary and secondary prevention of hypertension: A cross-sectional and longitudinal cohort study from NHANES analysis

**DOI:** 10.3389/fnut.2023.1129667

**Published:** 2023-03-14

**Authors:** Yanjun Song, Zhen'ge Chang, Kongyong Cui, Chenxi Song, Zhongxing Cai, Boqun Shi, Qiuting Dong, Kefei Dou

**Affiliations:** ^1^Cardiometabolic Medicine Center, Fuwai Hospital, National Center for Cardiovascular Diseases, Chinese Academy of Medical Sciences and Peking Union Medical College, Beijing, China; ^2^Cardiometabolic Medicine Center, State Key Laboratory of Cardiovascular Disease, Beijing, China; ^3^National Clinical Research Center for Cardiovascular Diseases, Beijing, China; ^4^Department of Respiratory Medicine, Civil Aviation General Hospital, Beijing, China

**Keywords:** hypertension, prevention, the MIND diet, dietary pattern, nutrition, prognosis

## Abstract

**Background:**

The Mediterranean-Dietary Approaches to Stop Hypertension for neurodegenerative delay (MIND) has been regarded as a novel healthy dietary pattern with huge benefits. However, its value in preventing and treating hypertension has not been investigated. The objective of this study is to investigate the impact of adhering to the MIND diet on the prevalence of hypertension in the entire population and long-term mortality in hypertensive patients.

**Methods:**

In this cross-sectional and longitudinal study, 6,887 participants consisting of 2,984 hypertensive patients in the National Health and Nutritional Examination Surveys were analyzed and divided into 3 groups according to the MIND diet scores (MDS; groups of MDS-low [<7.5], MDS-medium [7.5–8.0] and MDS-high [≥8.5]). In the longitudinal analysis, the primary outcome was all-cause death and the secondary outcome was cardiovascular (CV) death. Hypertensive patients received a follow-up with a mean time of 9.25 years (median time: 111.1 months, range 2 to 120 months). Multivariate logistics regression models and Cox proportional hazards models were applicated to estimate the association between MDS and outcomes. Restricted cubic spline (RCS) was used to estimate the dose–response relationship.

**Results:**

Compared with the MDS-low group, participants in the MDS-high group presented a significantly lower prevalence of hypertension (odds ratio [OR] 0.76, 95% confidence interval [CI] 0.58, 0.97, *p* = 0.040) and decreased levels of systolic blood pressure (*β* = −0.41, *p* = 0.033). Among hypertensive patients, 787 (26.4%) all-cause death consisting of 293 (9.8%) CV deaths were recorded during a 10-year follow-up. Hypertensive patients in the MDS-high group presented a significantly lower prevalence of ASCVD (OR = 0.71, 95% CI, 0.51, 0.97, *p* = 0.043), and lower risk of all-cause death (hazard ratio [HR] = 0.69, 95% CI, 0.58, 0.81, *p* < 0.001) and CV death (HR = 0.62, 95% CI, 0.46, 0.85, *p* for trend = 0.001) when compared with those in the MDS-low group.

**Conclusion:**

For the first time, this study revealed the values of the MIND diet in the primary and secondary prevention of hypertension, suggesting the MIND diet as a novel anti-hypertensive dietary pattern.

## 1. Introduction

Hypertension, known as one of the standard modifiable cardiovascular risk factors, greatly contributes to atherosclerotic cardiovascular disease (ASCVD) development and health burden worldwide (1). Investigations exploring dietary approaches with anti-hypertensive value have been extensively performed. In previous studies, the Dietary Approaches to Stop Hypertension (DASH) diet and the Mediterranean (MED) diets have been widely demonstrated to confer huge benefits in preventing hypertension (2, 3). Recently, the MED-DASH Intervention for Neurodegenerative Delay (MIND) diet, a promising dietary pattern designed from most of the components in the MED and DASH diets, attached great attention for its great protective values in cognitive performance (4). As for its components, the MIND diet emphasizes the consumption of whole grains, green leafy vegetables, olive oil, nuts, beans, berries, poultry, and seafood, and restricts the intake of fast-fried foods, sweets, butter, and margarine (5). In addition to the cognitive protection, benefits brought by the MIND diet are ongoingly revealed, such as preventing ASCVD (6), protecting physical function through strengthening muscles (7), reducing depression symptoms (8), and lowering the risk of breast cancer (9). Since the MIND diet is designed from the DASH and MED diets and has shown great therapeutic potential in numerous aspects, the value of adhering to the MIND diet in the prevention and treatment of hypertension raised interest. However, evidence for this aspect is scarce.

In this study, we analyzed 6,887 participants consisting of 2,984 hypertensive patients based on The U.S. National Health and Nutrition Examination Survey (NHANES), aiming to investigate the value of the MIND diet in the primary and secondary prevention of hypertension.

## 2. Materials and methods

### 2.1. Study design and participants

**Figure 1:**
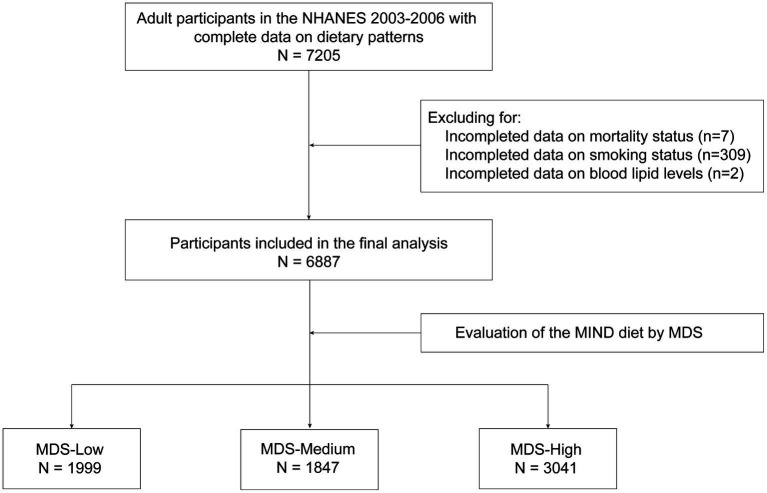
Flowchart of participant selection. MDS-Low, Low MIND score, the MIND diet score < 7.5; MDS-Mdeium, Medium MIND score, the MIND diet score ≥ 8 and < 8.5; MDS-High, High MIND score, the MIND diet score ≥ 8.5. NHANES, National Health and Nutrition Examination Survey; MIND diet, Mediterranean-DASH Diet Intervention for Neurodegenerative Delay diet; MDS, the MIND diet scores.

In this study, we utilized data from the 2 continuous cycles of NHANES from 2003 to 2006 because of no available questionnaire data on the MIND diet in the other years. Adult participants were included in this study, and the exclusion criterion was a lack of data on the components of the MIND diet. A total of 7,205 adult participants with complete data on dietary patterns were included initially. After excluding participants without information on smoking status (*n* = 309), mortality data (*n* = 7), and blood lipid levels (*n* = 2), we analyzed 6,887 participants (2,984 hypertensive patients) in the final analysis (Figure 1). Generally, 6,887 participants were ultimately included in this study. Participants in this study were allocated into 3 groups according to the MIND diet scores (MDS): groups of MDS-low (< 7.5), MDS-medium (7.5–8.0), and MDS-high (≥ 8.5). The optimal MDS cut-offs were defined as the tertiles of MDS in all participants. The main focus of this study is the value of the MIND diet in primary prevention (the association of MDS with the levels of blood pressure [BP] and the prevalence of hypertension in the entire population) and secondary prevention (the association of MDS with the prevalence of ASCVD, and the risk of all-cause death, and CV death in hypertensive patients) of hypertension.

Hypertension was defined as a self-reported medical history of high blood pressure, receiving antihypertensive drugs, or blood pressure measurement ≥140/90 mmHg (10), and ASCVD was defined as a series of coronary artery disease (CAD), heart attack, angina, congestive heart failure, stroke, and peripheral artery disease.

### 2.2. Dietary assessment

In this study, we analyzed each diet component relevant to the MIND diet according to the Food Frequency Questionnaire during the NHANES 2003 to 2006-year cycles. The food frequency questionnaire (FFQ) of this study was only conducted at baseline, and concrete information was presented in Supplementary Text S1. MDS was applicated to evaluate adherence to the MIND diet. The MIND diet includes 10 brain-healthy food groups (green leafy vegetables, other vegetables, nuts, berries, beans, whole grains, seafood, poultry, olive oil, and wine) and 5 unhealthy food groups (red meats, butter, and stick margarine, cheese, pastries and sweets, and fried/fast food). Olive oil consumption was scored 1 if identified by the participant as the primary oil usually used at home and 0 otherwise. For all other diet score components, we summed the frequency of consumption of each food item portion associated with that component and then assigned a concordance score of 0, 0.5, or 1. The total MDS was computed by summing over all 15 of the component scores, concrete information on the calculation of MDS was presented in Supplementary Table S1 (4).

### 2.3. Follow-up and outcomes

The primary outcome of this study was all-cause and the secondary outcome was CV death. Mortality status was ascertained with death certificate records by linkage to the National Death Index through December 31, 2019. The specific cause of death was determined based on the International Statistical Classification of Disease, Tenth Revision (ICD-10). CV death was defined as deaths from heart diseases (ICD-10 codes I00-I09, I11, I13, I20-I51) or cerebrovascular diseases (ICD-10 codes I60-I69). The follow-up time was calculated from the NHANES Mobile Examination Center (MEC) date to the date of death or end of follow-up (December 31, 2019), whichever came first. These final mortality statuses, follow-up time, and the underlying leading causes of death files are available for online access.^1^

### 2.4. Covariates

Covariates were collected at baseline (NHANES 2003–2004). Information on age, sex, race/ethnicity, smoking status, alcohol consumption, physical activity, and self-reported medical conditions were obtained through standardized questionnaires during in-home interviews by trained interviewers. Heights, weights, waist circumferences, blood pressures, and blood samples were collected from physical examinations at Mobile Examination Center (MEC) using standard protocols.

Race/ethnicity was categorized as non-Hispanic White people, non-Hispanic Black people, Mexican American people, and others. Smoking status was categorized as never (smoked less than 100 cigarettes in life), former (smoked more than 100 cigarettes in life but quit smoke now), and current (smoked more than 100 cigarettes in life and still smoke some days or every day). Physical activity was measured as the weekly minutes of moderate and vigorous activities multiplied by the metabolic equivalent (MET) level and divided into four categories: sedentary (without regular physical activity, MET-minutes/week = 0), insufficient (0 < MET-minutes/week <500), moderate (500 ≤ MET-minutes/week ≤1,000), and high (>1,000 MET-minutes/week) (10), Body mass index (BMI, kg/m^2^) was calculated as weight in kilograms divided by height in meters squared. Diabetes mellitus was defined as a self-reported medical history of diabetes, receiving oral hypoglycemic agents or insulin, fasting glucose level ≥ 126 mg/dl, or hemoglobin A1c (HbA1c) level ≥ 6.5% (11). Hyperlipidemia was defined as serum triglycerides (TG) ≥ 150 mg/L, total cholesterol (TC) ≥200 mg/dl, low-density lipoprotein cholesterol (LDL-C) ≥ 130 mg/dL, high-density lipoprotein cholesterol (HDL-C) ≤ 40 mg/dl in men or ≤ 50 mg/dL in women, or receiving medication for hyperlipidemia (12). The biochemical parameters, including TG, TC, HDL-C, LDL-C, and HbA1c were measured among partial participants who provided blood samples (95.7%, 6590/6887) at MEC.

### 2.5. Statistical analysis

As part of the NHANES complex sampling design, we utilized appropriate weights to ensure a representative sample of the US national population.^2^ The results of baseline characteristics were presented as weighted means ± standard error for continuous variables and frequency (weighted percentages) for categorical variables. We compared the differences among groups using ANOVA for continuous variables and χ2 tests for categorical variables. The percentages of missing data for covariates were lower than 5% (BMI [1.7%], and energy intake [5.0%]). Imputation with the median of each variable was used to incorporate all data for modeling.

The analysis mainly included three parts: (1) In the first part of our analysis, we made a cross-sectional analysis of the entire population to investigate the association of adhering to the MIND diet with the prevalence of hypertension and levels of BP, (2) Next, we focused on hypertensive patients and made a cross-sectional analysis to explore the association of adhering to the MIND diet with the prevalence of ASCVD, and (3) At last, we performed a longitudinal analysis (a 9.25-year clinical follow-up) for hypertensive patients with the outcomes of all-cause and cardiovascular death to explore the value of the MIND diet in the secondary prevention of hypertension.

The odd ratios (ORs) and 95% confidence intervals (CIs) for the association of MDS with the prevalence of hypertension and ASCVD were estimated using multi-variate Logistics regression models (cross-sectional analysis), and the hazard ratios (HRs) and 95% CIs for the association of MDS with the risk of all-cause death and CV death were explored using multivariate Cox proportional hazards models (longitudinal analysis). The correlation of MDS with levels of BP was estimated in the linear regression with the fully adjusted model. Restricted cubic spline (RCS) with 4 knots (5^th^, 35^th^, 65^th^, and 95^th^ percentiles) in the fully adjusted model was used to estimate the dose–response relationship between MDS and outcomes. Nonlinearity was tested using the likelihood ratio test. Apart from the crude model, we adjusted potential covariates progressively in the 3 models. Model 1 was adjusted for age (continuous), sex (male or female), and race/ethnicity (non-Hispanic White people, non-Hispanic Black people, Mexican American people, and other). Model 2 was further adjusted for all covariates in Model 1 and smoking status (never, former, and current), physical activity (sedentary, insufficient, moderate, and high), and BMI (< 25.0, 25.0–29.9, and ≥ 30.0 kg/m2). Model 3 (fully adjusted model) was further adjusted for all covariates in Model 2 and diabetes, hyperlipidemia, and energy intake.

Subgroup analysis was performed by age (< 60 or ≥ 60 years), sex (male or female), race/ethnicity (White people or non-White people), smoking status (never or former and current), BMI (<30.0 or ≥ 30.0 kg/m2), physical activity (sedentary and insufficient or moderate and high), and diabetes (yes or no), and examined the significance of multiplicative interaction terms between the stratification variables and MDS by the Wald test.

Sensitivity analyzes were conducted based on the fully adjusted model. Firstly, we excluded non-Hispanic Black participants because of the higher prevalence of mortality among non-Hispanic Black individuals. Secondly, we excluded Mexican American people and other participants because of the oversampled non-Hispanic participants. Thirdly, we excluded participants who died within 1 year of follow-up to minimize the potential reverse causation bias. Finally, we excluded participants with cerebral diseases because of the brain-protective effects of the MIND diet which might enlarge its protective effects on long-term mortality.

All analyzes were performed with *R* version 4.1.3 (*R* Foundation for Statistical Computing, Vienna, Austria) using the “survey” package. A 2-tailed value of *p* <0.05 was considered significant.

## 3. Results

### 3.1. Characteristics of the study population

Generally, 6,887 participants (age range:20 to 85 years old) were ultimately included in this study. In this study, MDS in all participants ranged from 4.5 to 13 points (Supplementary Table S2; Supplementary Figure S1). Table 1 showed the baseline characteristics of participants grouped by MDS. The overall weighted mean age of all participants was 47.13 years and 53.8% of them were female. Participants who better adhered to the MIND diet were older, more likely to be non-Hispanic white people, non-smokers, and have higher levels of DBP, physical activity, and less likely to combine cerebral diseases. The baseline characteristics of excluded participants with incomplete data were presented in Supplementary Table S3.

**Table 1:** Baseline characteristics of all participants based on the MIND diet score.

Characteristics	MDS tertile				*p* trend
	Total (*N* = 6,887)	MDS-L (*N* = 1999)	MDS-M (*N* = 1847)	MDS-H (*N* = 3,041)	
Age (years)	47.13 ± 0.45	42.97 ± 0.52	47.37 ± 0.57	49.66 ± 0.61	< 0.001
Sex, *n* (%)					< 0.001
Male	3,181 (46.19)	1,051 (52.29)	876 (49.14)	1,254 (39.13)	
Female	3,706 (53.81)	948 (47.71)	971 (50.86)	1787 (60.87)	
Race/ethnicity, *n* (%)					< 0.001
Non-Hispanic White people	3,809 (55.31)	1,080 (70.53)	993 (70.51)	1736 (75.53)	
Non-Hispanic Black people	1,333 (19.36)	478 (15.09)	369 (12.02)	486 (8.67)	
Mexican American people	1,272 (18.47)	318 (7.11)	353 (8.44)	601 (8.20)	
Others	473 (6.87)	123 (7.27)	132 (9.03)	218 (7.60)	
Smoking status, *n* (%)					< 0.001
Never	3,526 (51.2)	923 (43.95)	964 (49.76)	1,639 (53.92)	
Former	1920 (27.88)	478 (20.01)	485 (25.40)	957 (30.43)	
Current	1,441 (20.92)	598 (36.04)	398 (24.84)	445 (15.65)	
BMI (kg/m2), *n* (%)					0.07
<25.0	2082 (30.71)	610 (35.08)	531 (32.15)	941 (33.80)	
25.0–29.9	2,356 (34.75)	639 (29.51)	635 (34.03)	1,082 (34.85)	
≥30.0	2,341 (34.53)	717 (35.41)	653 (33.82)	971 (31.35)	
Physical activity, *n* (%)					< 0.001
Sedentary	1774 (25.76)	610 (23.94)	504 (19.68)	660 (15.03)	
Insufficient	2,621 (38.06)	728 (39.66)	726 (43.71)	1,167 (41.41)	
Moderate	1,129 (16.39)	302 (17.10)	289 (16.32)	538 (19.04)	
High	1,363 (19.79)	359 (19.30)	328 (20.29)	676 (24.52)	
MDS (score)	8.16 ± 0.05	6.45 ± 0.02	7.75 ± 0.01	9.49 ± 0.03	< 0.001
DBP (mmHg)	54.61 ± 0.31	51.75 ± 0.60	53.74 ± 0.42	56.93 ± 0.52	< 0.001
SBP (mmHg)	123.26 ± 0.45	122.41 ± 0.55	123.71 ± 0.74	123.53 ± 0.55	0.23
Hypertension, n (%)	2,984 (43.33)	846 (36.03)	841 (40.34)	1,297 (38.27)	0.21
Diabetes, *n* (%)	1,021 (14.83)	304 (11.01)	277 (11.07)	440 (10.78)	0.96
Hyperlipidemia, n (%)	4,954 (71.93)	1,428 (69.18)	1,347 (71.10)	2,179 (69.96)	0.68
Cerebral diseases, *n* (%)	287 (4.17)	96 (3.82)	83 (2.80)	108 (2.15)	0.003
TG (mg/dL)	142.74 ± 2.53	150.65 ± 5.04	141.39 ± 4.38	138.86 ± 4.20	0.21
HbA1c (%)	70.47 ± 0.28	70.70 ± 0.54	70.32 ± 0.50	70.41 ± 0.38	0.87
Fast blood glucose (mg/dL)	5.47 ± 0.02	5.45 ± 0.03	5.50 ± 0.03	5.46 ± 0.02	0.48
Waist circumference (cm)	97.53 ± 0.45	98.50 ± 0.59	97.89 ± 0.62	96.71 ± 0.63	0.08
Energy intake (Kcal)	2117.02 ± 15.62	2219.03 ± 28.08	2093.35 ± 21.63	2065.08 ± 23.42	< 0.001

Data are presented as weighted means ± SEs for continuous variables and unweighted numbers (weighted percentages) for categorical variables.

MDS-L, Low MIND score, the MIND diet score < 7.5; MDS-M, Medium MIND score, the MIND diet score ≥ 8 and < 8.5; MDS -H, High MIND score, the MIND diet score ≥ 8.5.

MDS, the MIND diet scores; BMI, body mass index; SBP, systolic blood pressure; DBP, diastolic blood pressure; TG, triglyceride; HbA1c, glycosylated hemoglobin, type A1C.

### 3.2. Association of adhering to the MIND diet with the prevalence of hypertension in the whole population

**Figure 2:**
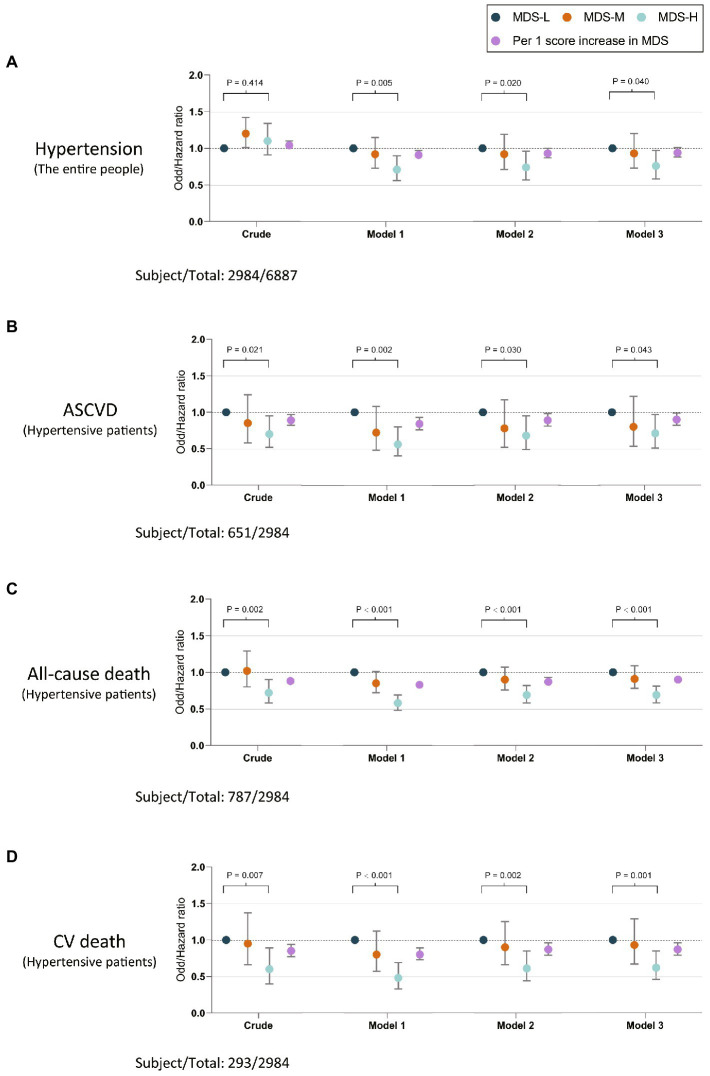
Odds/hazard ratios and 95%CI for the associations of MDS with the risk of hypertension in entire participants **(A)**, and the risk of ASCVD **(B)**, all-cause death **(C)**, and CV death **(D)**. MDS-L, Low MIND score, the MIND diet score < 7.5; MDS-M, Medium MIND score, the MIND diet score ≥ 8 and < 8.5; MDS -H, High MIND score, the MIND diet score ≥ 8.5. ASCVD, atherosclerotic cardiovascular disease; CV, cardiovascular; MIND diet, Mediterranean-DASH Diet Intervention for Neurodegenerative Delay diet; MDS, the MIND diet scores.

**Figure 3:**
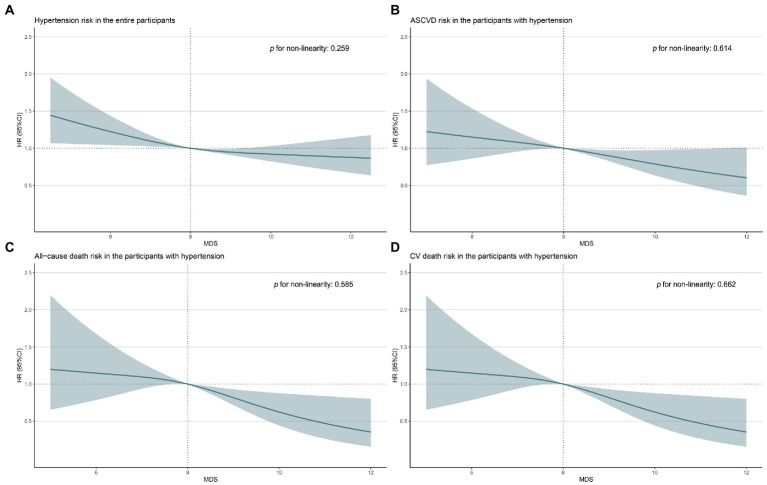
Restricted cubic spline models for the associations of MDS with the risk of hypertension in the entire participants **(A)**, and the risk of ASCVD **(B)**, all-cause death **(C)**, and CV death **(D)** in hypertensive patients. ASCVD, atherosclerotic cardiovascular disease; CV, cardiovascular; MIND diet, Mediterranean-DASH Diet Intervention for Neurodegenerative Delay diet; MDS, the MIND diet scores.

Table 2 and Figure 2A presented the logistic regression results of the association of adhering to the MIND diet with the prevalence of hypertension in 3 different models. Although no significant association was found in the crude model, results in other models presented that the MDS-high group showed a significantly lower prevalence of hypertension compared with participants in the low MDS group (model 3, HR 0.76, 95% CI 0.58, 0.97, *p* = 0.040). Besides, per one-score increase in MDS was shown to be associated with a 9% lower prevalence of hypertension (model 2, HR 0.91, 95% CI, 0.86, 0.97) (Table 2), and RCS showed a linear association of MDS with the prevalence of hypertension (*p* for non-linearity = 0.259) (Figure 3A).

**Table 2:** Logistic regression analysis for the risk of hypertension according to the MIND diet score in the whole population.

Model	Per one-score increases in MDS OR (95% CI)	OR (95% CI)			*p* trend
		MDS-L	MDS-M	MDS-H	
Crude	1.04 (0.99, 1.10)	1.00	1.20 (1.01, 1.42)	1.10 (0.91, 1.34)	0.414
Model 1	0.91 (0.86, 0.97)	1.00	0.92 (0.73 1.15)	0.71 (0.56, 0.90)	0.005
Model 2	0.93 (0.87, 1.00)	1.00	0.92 (0.71, 1.19)	0.74 (0.57, 0.96)	0.020
Model 3	0.94 (0.88, 1.01)	1.00	0.93 (0.73, 1.20)	0.76 (0.58, 0.97)	0.040

Model 1, adjusted for age, sex, and race/ethnicity.

Model 2, further adjusted (from Model 1) for smoking status, BMI, and physical activity.

Model 3, further adjusted (from Model 2) for diabetes, dyslipidemia, and energy intake.

MDS-L, Low MIND score, the MIND diet score < 7.5; MDS-M, Medium MIND score, the MIND diet score ≥ 8 and < 8.5; MDS -H, High MIND score, the MIND diet score ≥ 8.5.

MIND, the Mediterranean-DASH Diet Intervention for Neurodegenerative Delay diet score; MDS, the MIND diet scores; OR, odds ratios; CIs, confidence intervals.

Linear regression analysis presented that MDS was inversely correlated with the levels of SBP (*β* = −0.41, *p* = 0.033) in the whole population (Supplementary Table S5).

### 3.3. Association of adhering to the MIND diet on prevalence of ASCVD and BP levels in hypertensive patients

The baseline characteristics of hypertensive participants in this study were presented in online Supplemental files (Supplementary Table S4), and 641 (21.5%) of them combined ASCVD. Table 3 showed that hypertensive participants in the MDS-high group presented a significantly lower prevalence of ASCVD (model 3, OR = 0.80, 95% CI, 0.51, 0.97, *p* for trend = 0.043) compared with the MDS-low group in all models (Figure 2B). Moreover, per one-score increase in MDS was found to be associated with a 10% lower prevalence of ASCVD (HR = 0.90, 95% CI, 0.82, 0.99) (Table 3), and RCS showed a linear association of MDS with the prevalence of ASCVD (*p* for non-linearity = 0.614) in hypertensive participants (Figure 3B). No significant correlation was found between MDS and BP levels in hypertensive patients (Supplementary Table S5).

**Table 3:** Logistic regression analysis for the risk of ASCVD and Cox regression analysis for all-cause and cardiovascular mortality according to the MIND diet score among hypertensive patients.

Model	Per one-score increases in MDS OR/HR (95% CI)	ORs/HRs (95% CI)			*p* trend
		MDS-L	MDS-M	MDS-H	
*ASCVD*					
Number of ASCVD/totals	641/2984	203/846	183/841	255/1297	
Crude	0.89 (0.82, 0.97)	1.00	0.85 (0.58, 1.24)	0.70 (0.52, 0.95)	0.021
Model 1	0.84 (0.76, 0.93)	1.00	0.72 (0.48 1.08)	0.56 (0.40, 0.80)	0.002
Model 2	0.89 (0.81, 0.98)	1.00	0.78 (0.52, 1.17)	0.68 (0.49, 0.95)	0.030
Model 3	0.90 (0.82, 0.99)	1.00	0.80 (0.53, 1.22)	0.71 (0.51, 0.97)	0.043
*All-cause mortality*					
Number of deaths/totals	787/2984	254/846	234/841	299/1297	
Crude	0.88 (0.83, 0.93)	1.00	1.02 (0.80, 1.29)	0.72 (0.58, 0.90)	0.002
Model 1	0.83 (0.79, 0.87)	1.00	0.85 (0.72 1.01)	0.58 (0.48, 0.69)	<0.001
Model 2	0.87 (0.83, 0.93)	1.00	0.90 (0.76, 1.07)	0.69 (0.58, 0.82)	<0.001
Model 3	0.90 (0.86, 0.95)	1.00	0.91 (0.78, 1.09)	0.69 (0.58, 0.81)	<0.001
*CV mortality*					
Number of deaths/totals	293/2984	97/846	88/841	108/1297	
Crude	0.85 (0.77, 0.94)	1.00	0.95 (0.66, 1.37)	0.60 (0.40, 0.89)	0.007
Model 1	0.80 (0.73, 0.89)	1.00	0.80 (0.57, 1.12)	0.48 (0.33, 0.69)	<0.001
Model 2	0.87 (0.79, 0.96)	1.00	0.90 (0.66, 1.25)	0.61 (0.44, 0.85)	0.002
Model 3	0.87 (0.79, 0.96)	1.00	0.93 (0.67, 1.29)	0.62 (0.46, 0.85)	0.001

Model 1, adjusted for age, sex, and race/ethnicity.

Model 2, further adjusted (from Model 1) for smoking status, BMI, and physical activity.

Model 3, further adjusted (from Model 2) for diabetes, dyslipidemia, and energy intake.

MDS-L, Low MIND score, the MIND diet score < 7.5; MDS-M, Medium MIND score, the MIND diet score ≥ 8 and < 8.5; MDS -H, High MIND score, the MIND diet score ≥ 8.5.

ASCVD, atherosclerotic cardiovascular disease; CV, cardiovascular; MDS, the MIND diet scores; ORs, odd ratio; HRs, hazard ratios; CIs, confidence intervals; MDS, the MIND diet score, the Mediterranean-DASH Diet Intervention for Neurodegenerative Delay diet score; BMI, body mass index.

Besides, the inverse association of MDS with the prevalence of ASCVD was significant in the entire population (Supplementary Table S6), but not in participants without hypertension (Supplementary Table S7).

### 3.4. Association of adhering to the MIND diet with risk of all-cause death and CV death in hypertensive patients

During the follow-up, a total of 787 (26.4%) all-cause deaths and 293 (9.8%) CV deaths in hypertensive patients were recorded. As is shown in Table 3, Compared with participants in the low MDS group, hypertensive patients in the MDS-high group showed a significantly lower risk of all-cause death (model 3, HR = 0.69, 95% CI, 0.58, 0.81, *P* for trend <0.001) and CV death (model 3, HR = 0.62, 95% CI, 0.46, 0.85, *P* for trend = 0.001) in the all models (Figures 2C,D). Per one-score increase in MDS was associated with a 10% lower risk of all-cause death (HR = 0.90, 95% CI, 0.86, 0.95) and a 13% lower risk of CV death (HR = 0.87, 95% CI, 0.79, 0.96) (Table 3). Besides, RCS showed linear associations of MDS with the risk of all-cause death (*p* for non-linearity = 0.585) and CV death (*p* for non-linearity = 0.662) in hypertensive participants (Figures 3C,D).

Kaplan–Meier curves for all-cause mortality and CV mortality of 3 groups among hypertensive patients were further performed (Supplementary Figure S2). Consistently, groups with higher MDS showed a significantly lower risk of all-cause death (Log-rank test, *p* < 0.001) and CV death (Log-rank test, *p* = 0.014).

Besides, the inverse associations of MDS with the risk of all-cause death and CV death were significant in the entire population (Supplementary Table S6). As for participants without hypertension, the inverse associations of MDS with the risk of all-cause death, but not CV death, was significant (Supplementary Table S7).

### 3.5. Verification of results

Subgroup analyzes for the prevalence of hypertension in the whole population (Supplementary Table S8; Supplementary Figure S3), the prevalence of ASCVD (Supplementary Table S9; Supplementary Figure S4), and the risk of all-cause death (Supplementary Table S10; Supplementary Figure S5), and CV death (Supplementary Table S11; Supplementary Figure S6) in hypertensive patients were presented in supplemental files. Analyzes were stratified by age (<60 or ≥ 60 years), sex (male or female), race/ethnicity (non-Hispanic White people or other), smoking status (never or former/current), hypertension (yes or no), diabetes (yes or no), BMI (<30 or ≥ 30 kg/m^2^) and physical activity (sedentary/insufficient or moderate/high). Results in subgroup analyzes did not change. Specifically, significant interactions were found in the subgroup analysis of age for the risk of all-cause death, the subgroup analysis of sex for the prevalence of ASCVD, and all-cause death, and the subgroup analysis of race for the risk of CV death.

When it came to the sensitivity analysis for the risk of all-cause death and CV death in hypertensive participants (Supplementary Table S12), the results remained consistent after excluding Hispanic participants, Mexican American and other participants, individuals who died within 1 year of follow-up, and participants with cerebral diseases.

To further validate the results above, we compared subjects with the mean score of each MDS category and found that the results were consistent (Supplementary Table S13). Besides, we also explored the protective effect of each food component in Supplementary Table S14. Results showed that the points were largely from restricted intake of butter and red meat. High intakes of fish, green leafy vegetables, nuts, and poultry, limited consumption of red meat, and proper intake of wine were the main protective contributors.

## 4. Discussion

In this cross-sectional and longitudinal study, a total of 6,887 participants consisting of 2,984 hypertensive patients were ultimately included. The main findings of this study include (1) better adherence to the MIND diet is associated with a lower prevalence of hypertension in the whole population, (2) hypertensive patients who adhered better to the MIND diet presented a lower prevalence of ASCVD, and a lower risk of all-cause death, and CV death, and (3) The inverse associations of MDS with the prevalence of hypertension, ASCVD, and the risk of all-cause death, and CV death all presented as linear relationships, and per 1-score increase in MDS was shown to significantly reduce the risk above. To date, this study documented the protective value of adhering to the MIND diet in both primary and secondary prevention of hypertension for the first time.

Investigations for anti-hypertensive dietary patterns have nowadays been widely performed. In previous studies, the DASH and MED diets have been revealed to confer great value in the prevention of hypertension, since numerous randomized control trials (RCT) reported that the MED and DASH diets significantly decreased both SBP and DBP in the whole population (2, 3). The MIND diet was initially designed based on the dietary components of the MED and DASH diets, including the great emphasis on natural plant foods and restricted consumption of animal and high saturated fat foods (4). However, the anti-hypertensive value of the MIND diet has not been investigated so far. For the first time, this study revealed that better adherence to the MIND diet was associated with decreased SBP and lower prevalence of hypertension in the whole population, documenting the significant value of the MIND diet in the primary prevention of hypertension.

Another major finding of this study is firstly revealing the values of the MIND diet in the secondary prevention of hypertension. Numerous studies have confirmed the therapeutic benefits in hypertensive patients who adhered to the DASH diet (3, 13–15) and the MED diet (2, 16). As for the MIND diet, its benefits of lowering long-term all-cause mortality in old participants have been reported recently (17), however, no research explored the therapeutic value of the MIND diet among hypertensive patients. In this study, we focused on patients with hypertension and revealed the improved prognosis in those with better adherence to the MIND diet. In addition to all-cause mortality, the cardioprotective potential of the MIND diet was recently discussed. In a current prospective cohort study with 2,863 participants, Mahdieh et al. revealed a significant inverse relationship between MDS and CVD (comprised of CAD, stroke, and CV mortality) risk (6). Besides, a recent case–control study focused on patients with stroke also presented similar results (18). Consistent with previous studies, a significantly lower risk of ASCVD and CV death was also reported in the entire population and hypertensive patients who better adhered to the MIND diet, further supporting that the MIND diet was a cardioprotective dietary pattern and concreting the therapeutic value of the MIND diet in the secondary prevention of hypertension.

In the subgroup analyzes of this study, we reported that the protective anti-hypertensive impact of the MIND diet was more significant in old people, females, and non-white people. These results indicated the protective roles of the MIND diet might be various in different people. Thus, future studies are also expected to compare the beneficial impact of adhering to the MIND diet on participants with different age groups, sex, and races to further illuminate the anti-hypertensive roles of the MIND diet.

In the components of MIND diets, we found that MDS was largely coming from a restricted intake of butter and red meat. High intakes of fish, green leafy vegetables, nuts, and poultry, limited consumption of red meat, and proper intake of wine were the main protective contributors. In previous studies, the high intake of fish with huge sources of α-linolenic acid and marine omega-3 fatty acids (19), and high consumption of whole grains (20), olive oil (21), beans (22), and nuts (23), which were also emphasized in the DASH or MED diet, have been demonstrated as great anti-hypertension dietary components in previous studies. In addition, the MIND diet uniquely emphasized the consumption of berries and green leafy vegetables for dementia prevention, and these components also presented anti-hypertensive effects. In a rigorous investigation performed on hypertensive rats, Melissa et al. reported that the 6 week consecutive consumption of an experimental diet containing 4% green leafy vegetables significantly decreased SBP (24). Moreover, the consumption of berries was also indicated to hold great therapeutic potential in both the prevention and treatment of hypertension, as numerous recent clinical trials and RCT reported that the consumption of berries brought a significant reduction in levels of SBP and DBP regardless of the combination of hypertension (25–27). As for the mechanisms, great sources of quercetin, flavonoid, and folate from green leafy vegetables and berries were shown to be the main contributors through great anti-oxidative effects and vascular protection (28–30). As for the red meat, poultry, and wine intake, their associations with hypertension were still controversial, our results need to be further validated in future studies.

In the subgroup analysis, we found that the protective effect of the MIND diet was more significant in females. This conclusion was also reported by a recent study that focused on the relationship between the MIND diet and the risk of dementia, as the result showed that MIND adherence contributed to a decrease in the risk of dementia in females but not males (31). Besides, a more significant association between adherence to the MIND diet and a decrease in mortality among olde people was reported in the current study. Although there is no study comparing the protective effects of the MIND diet between old people and young people before, scholars have previously focused on old people and determined that closer adherence to the MIND diet is significantly associated with lower all-cause mortality (17). The interaction between race and the MIND diet has not been discussed previously, and the current study reported that the MIND diet-related reduction in the risk of CV death was more significant in white people for the first time. This conclusion was warranted to be further validated in future studies focusing on white people.

The main strength of this study is the first illustration of the impact of adhering to the MIND diet on the prevalence of hypertension in the whole population, and the prevalence of ASCVD and prognosis in hypertensive patients. However, there were several limitations in this study. Firstly, the association of adhering to the MIND diet with the prevalence of hypertension and ASCVD was investigated in cross-sectional analyzes, which might not have identified robust causal inferences. Secondly, the diagnosis of ASCVD was based on questionnaires without medical records, laboratory tests, or imaging, which might cause misdiagnosis. Thirdly, the 24 h dietary recall data was not applied in this study for the reason that these data mostly presented as “g/day” but not “serving/week” which is not suitable for the calculation of the MIND diet scores. Fourthly, the non-Hispanic participants were oversampled in the NHANES database. To decrease the bias from ethnicity, we made the related subgroup and sensitivity analysis. Nevertheless, it is still worthy to be validated in a population with proper percentages of ethnicity. Fifthly, the intake of each component of the MIND diet was collected based on a questionnaire without correction from specialists and continuous follow-up, which might contribute to bias. At last, the small or moderate sample size of this study limited the strength of the conclusions. Therefore, future exploration for the association of the MIND diet with hypertension is expected to be performed in large cohort studies or RCTs.

## 5. Conclusion

In conclusion, this study focused on the whole population and hypertensive patients and revealed the therapeutic potential of the MIND diet in the primary and secondary prevention of hypertension. These results documented the MIND diet as a novel anti-hypertensive dietary pattern for the first time.

## Data availability statement

Publicly available datasets were analyzed in this study. This data can be found here: https://www.cdc.gov/nchs/nhanes/index.htm.

## Ethics statement

Ethical review and approval was not required for the study on human participants in accordance with the local legislation and institutional requirements.

## Author contributions

KD and YS: conceptualization. QD, YS, and ZCh: methodology. YS and ZCh: software and formal analysis. YS and KC: validation. YS, ZCh, KC, and ZCa: investigation. KD and QD: resources. ZCh: data curation. YS, ZCh, KC, CS, and BS: writing—original draft preparation. QD and KD: writing—review and editing. YS: visualization. KD: supervision, project administration, and funding acquisition. All authors contributed to the article and approved the submitted version.

## Funding

This work was supported by CAMS Innovation Fund for Medical Sciences (CIFMS) (grant no. 2021-I2M-1-008).

## Conflict of interest

The authors declare that the research was conducted in the absence of any commercial or financial relationships that could be construed as a potential conflict of interest.

## Publisher’s note

All claims expressed in this article are solely those of the authors and do not necessarily represent those of their affiliated organizations, or those of the publisher, the editors and the reviewers. Any product that may be evaluated in this article, or claim that may be made by its manufacturer, is not guaranteed or endorsed by the publisher.

## Footnotes

^1^https://ftp.cdc.gov/pub/Health_Statistics/NCHS/datalinkage/linked_mortality

^2^https://wwwn.cdc.gov/nchs/nhanes/tutorials/module3.aspx
