# Supplementary material for: The value of the MIND diet in the primary and secondary prevention of hypertension: A cross-sectional and longitudinal cohort study from NHANES analysis
Source: Front Nutr. 2023 Mar 14;10:1129667. doi: 10.3389/fnut.2023.1129667 (PMC10043250; doi:10.3389/fnut.2023.1129667)
Supplement: Supplementary file 1 [file Data_Sheet_1.docx]

**SUPPLEMENTARY DATA**

**Better adherence to the Mediterranean - Dietary Approaches to Stop Hypertension intervention for neurodegenerative delay (MIND) diet is associated with a lower prevalence of hypertension in the whole population and improved prognosis in hypertensive patients**

Yanjun Song, MD; Zhen'ge Chang, MSc; Kongyong Cui, MD; Chenxi Song, MD; Zhongxing Cai, MD; Boqun Shi, MD; Qiuting Dong, MD; Kefei Dou, MD PhD

**Online Supplementary Material**

1. **Text S1.** Introduction to the food frequency questionnaire
2. **Table S1.** The calculation for the MIND scores
3. **Table S2.** The MIND scores in the included participants.
4. **Table S3.** Comparisons of baseline characteristics between participants with complete and incomplete data.
5. **Table S4.** Baseline characteristics of participants with hypertension based on the MIND diet score
6. **Table S5.** Liner relationship between the blood pressure and the MIND diet score among the entire population and hypertensive patients.
7. **Table S6.** Logistic regression analysis for the prevalence of ASCVD and Cox regression analysis for the risk of all-cause and cardiovascular mortality according to the MIND diet score in the entire population.
8. **Table S7.** Logistic regression analysis for the prevalence of ASCVD and Cox regression analysis for the risk of all-cause and cardiovascular mortality according to the MIND diet score among participants without hypertension.
9. **Table S8.** Subgroup analyses of the associations between MIND diet score and hypertension among the entire participants.
10. **Table S9.** Subgroup analyses of the associations between MIND diet score and the prevalence of ASCVD among patients with hypertension.
11. **Table S10.** Subgroup analyses of the associations between MIND diet score and all-cause mortality among patients with hypertension
12. **Table S11.** Subgroup analyses of the associations between MIND diet score and CV mortality among patients with hypertension.
13. **Table S12.** Sensitivity Analyses of the associations between MIND diet score and all-cause/CV mortality among hypertensive patients.
14. **Table S13**. Logistic regression analysis for the prevalence of hypertension and ASCVD and Cox regression analysis for the risk of all-cause and cardiovascular mortality according to the mean MIND diet score of each MDS category.
15. **Table S14**. Associations of individual food components of the MIND diet with the prevalence of hypertension, the prevalence of ASCVD, the risk of all-cause and cardiovascular mortality.
16. **Figure S1.** The MIND diet scores of included participants grouped by hypertension status.
17. **Figure S2.** Kaplan-Meier curves for the all-cause and CV mortality in groups of MDS-L, MDS-M, and MDS-H in hypertensive patients.
18. **Figure S3.** Subgroup analysis for the prevalence of hypertension in the entire population.
19. **Figure S4.** Subgroup analysis for the prevalence of ASCVD in hypertensive patients.
20. **Figure S5.** Subgroup analysis for the risk of all-cause death in hypertensive patients.
21. **Figure S6.** Subgroup analysis for the risk of CV death in hypertensive patients.

**Text S1.** **Introduction to the food frequency questionnaire**

The food frequency questionnaire (FFQ) was used to collect information on the frequency of food consumption during the past 12 months, which provides information on the frequency covariates on food intake. The calculation of the MIND scores was based on the data of the frequency of food intake in the FFQ such as “How often did you drink wine or wine coolers?”, and the options of this question including never, 1 time per month or less, 2-3 times per month, 1-2 times per week, etc. The National Health and Nutrition Examination Survey (NHANES) FFQ questionnaire was developed by the National Institutes of Health, National Cancer Institute (NCI), which contains several types of questions. There are 151 frequency questions (this includes two frequency sub-questions querying intake over two seasons for questions such as “How often drink tomato juice?” etc.). The FFQ also asks about the proportion (reported as fractions) of the time certain types of foods were eaten over the past 12 months such as sugar-free soft drinks, whole grain foods, and light, low-fat or fat-free varieties of foods. In addition, quality control programs and manual verification checks were used to verify the completeness and accuracy of data (1, 2).

**Supplementary Tables**

Table S1. The calculation for the MIND scores

| Scores | 0 | 0.5 | 1 |
| --- | --- | --- | --- |
| Green leafy vegetables | ≤2 servings/wk | > 2 to <6 servings/wk | ≥6 servings/wk |
| Other vegetables | <5 servings/wk | 5 – <7 servings/wk | ≥1 serving/day |
| Berries | <1 serving/wk | 1 serving/wk | ≥2 servings/wk |
| Nuts | <1 serving/mo | 1 serving/mo – <5 servings/wk | ≥5 servings/wk |
| Olive Oil | Not primary oil |  | Primary oil used |
| Butter, Margarine | >2 T/d | 1–2 T/d | <1 T/d |
| Cheese | 7+ servings/wk | 1–6 servings/wk | < 1 serving/wk |
| Whole Grains | <1 serving/d | 1–2 servings/d | ≥3 servings/d |
| Fish (not fried) | Rarely | 1–3 meals/mo | ≥1 meal/wk |
| Beans | <1 meal/wk | 1–3 meals/wk | >3 meals/wk |
| Poultry (not fried) | <1 meal/wk | 1 meal/wk | ≥2 meals/wk |
| Red meat and products | 7+ meals/wk | 4–6 meals/wk | < 4 meals/wk |
| Fast fried foods | 4+ times/wk | 1–3 times/wk | <1 time/wk |
| Pastries & Sweets | 7+ servings/wk | 5 −6 servings/wk | <5 servings/wk |
| Wine | >1 glass/d or never | 1 glass/mon – 6 glasses/wk | 1 glass/d |
| Total scores |  |  | 15 |

Green leafy vegetables: kale, collards, greens; spinach; lettuce/tossed salad.

Other vegetables: green/red peppers, squash, cooked carrots, raw carrots, broccoli, celery, potatoes, peas or lima beans, potatoes, tomatoes, tomato sauce, string beans, beets, corn, zucchini/summer squash/eggplant, coleslaw, potato salad.

Berries: strawberries.

Fish (not fried): tuna sandwich, fresh fish as main dish; not fried fish cakes, sticks, or sandwiches

Beans: beans, lentils, soybeans

Poultry (not fried): chicken or turkey sandwich, chicken or turkey as main dish and never eat fried at home or away from home

Red meat and products: cheeseburger, hamburger, beef tacos/burritos, hot dogs/sausages, roast beef or ham sandwich, salami, bologna, or other deli meat sandwich, beef (steak, roast) or lamb as main dish, pork or ham as main dish, meatballs or meatloaf

Fast fried foods: How often do you eat fried food away from home (like French fries, chicken nuggets)

Pastries & Sweets: biscuit/roll, poptarts, cake, snack cakes/twinkies, Danish/sweet rolls/pastry, donuts, cookies, brownies, pie, candy bars, other candy, ice cream, pudding, milkshakes/frappes

Table S2. The MIND scores in the included participants

| Participants | MDS | MIND diet scores | | | | | | | | | | |
| --- | --- | --- | --- | --- | --- | --- | --- | --- | --- | --- | --- | --- |
|  | Mean ± SD | 3.5-4.0 | 4.5-5.0 | 5.5-6.0 | 6.5-7.0 | 7.5-8.0 | 8.5-9.0 | 9.5-10.0 | 10.5-11.0 | 11.5-12.0 | 12.5-13.0 | 13.5 |
| Total, n (%) | 8.14±1.44 | 3 (0.02) | 34 (0.80) | 563 (8.59) | 1399 (19.58) | 1847 (25.89) | 1539 (22.26) | 924 (13.56) | 424 (7.07) | 133 (1.94) | 20 (0.27) | 1 (0.01) |
| Hypertension, n (%) | 8.14±1.42 | 1 (0.01) | 14 (0.85) | 222 (5.76) | 609 (25.82) | 841 (27.37) | 662 (13.37) | 392 (13.00) | 171 (6.95) | 62 (2.53) | 10 (0.41) | 0 (0.00) |
| Non-hypertension, n (%) | 8.13±1.45 | 2 (0.01) | 20 (0.76) | 341 (9.52) | 800 (19.69) | 1006 (24.98) | 877 (22.20) | 532 (13.91) | 253 (7.15) | 71 (1.58) | 341 (0.19) | 1 (0.01) |

Abbreviations: MDS, MIND diet score.

Table S3. Comparisons of baseline characteristics between participants with complete and incomplete data

| Characteristics | Total (N=7205) | Data status | | *P* value | |  |
| --- | --- | --- | --- | --- | --- | --- |
|  |  | Complete (N=6887) | Incomplete (N=318) | |  | |
| Age (years) | 46.67±0.46 | 47.13±0.45 | 21.15±0.77 | | < 0.001 | |
| Sex, n (%) |  |  |  | | 0.72 | |
| Male | 3893 (54.03) | 3706 (54.47) | 187 (56.31) | |  | |
| Female | 3312 (45.97) | 3181 (45.53) | 131 (43.69) | |  | |
| Race/ethnicity, n (%) |  |  |  | | 0.1 | |
| Non-Hispanic White People | 3916 (54.35) | 3809 (72.78) | 107 (66.28) | |  | |
| Non-Hispanic Black People | 1438 (19.96) | 1333 (11.40) | 105 (18.91) | |  | |
| Mexican American People | 1358 (18.85) | 1272 (7.95) | 86 (8.72) | |  | |
| Others | 493 (6.84) | 473 (7.88) | 20 (6.10) | |  | |
| BMI (kg/m^2^), n (%) |  |  |  | | < 0.001 | |
| <25.0 | 2240 (31.58) | 2082 (33.75) | 158 (55.09) | |  | |
| 25.0–29.9 | 2438 (34.38) | 2356 (33.09) | 82 (24.30) | |  | |
| ≥30.0 | 2414 (34.04) | 2341 (33.17) | 73 (20.62) | |  | |
| Physical activity, n (%) |  |  |  | | 0.03 | |
| Sedentary | 1822 (25.29) | 1774 (18.82) | 48 (12.05) | |  | |
| Insufficient | 2764 (38.36) | 2621 (41.50) | 143 (40.73) | |  | |
| Moderate | 1176 (16.32) | 1129 (17.78) | 47 (13.18) | |  | |
| High | 1443 (20.03) | 1363 (21.91) | 80 (34.04) | |  | |
| SBP (mmHg) | 123.07±0.43 | 123.26±0.45 | 113.00±1.42 | | < 0.001 | |
| DBP (mmHg) | 70.37±0.28 | 70.47±0.28 | 65.03±1.40 | | < 0.001 | |
| Diabetes, n (%) | 1023 (14.2) | 1021 (10.92) | 2 (0.29) | | < 0.001 | |
| Hypertension, n (%) | 3013 (41.82) | 2984 (38.16) | 29 (11.25) | | < 0.001 | |
| HbA1c (%) | 5.46±0.02 | 5.47±0.02 | 5.04±0.03 | | < 0.001 | |
| Fast blood glucose (mg/dL) | 5.64±0.04 | 5.65±0.04 | 5.03±0.06 | | < 0.001 | |
| Waist circumference (cm) | 97.36±0.45 | 97.53±0.45 | 87.41±0.88 | | < 0.001 | |
| Energy intake (Kcal) | 2127.74±15.66 | 2126.53± 16.28 | 2197.74±101.67 | | 0.51 | |

Data are presented as weighted means ± SEs for continuous variables and unweighted numbers (weighted percentages) for categorical variables.

MDS-L, Low MIND score, the MIND diet score <7.5; MDS-M, Medium MIND score, the MIND diet score ≥8 and <8.5; MDS -H, High MIND score, the MIND diet score ≥8.5.

Abbreviations: MDS, the MIND diet scores; BMI, body mass index; SBP, systolic blood pressure; DBP, diastolic blood pressure; HbA1c, glycosylated hemoglobin, type A1C.

Table S4. Baseline characteristics of participants with hypertension based on the MIND diet score

| Characteristics | Total (N=2984) | MDS tertile | | | *P* trend |
| --- | --- | --- | --- | --- | --- |
|  |  | MDS-L (N=845) | MDS-M (N=841) | MDS-H (N=1298) |  |
| Age (years) | 57.40±0.68 | 54.10±1.06 | 57.56±0.80 | 59.30±0.76 | < 0.0001 |
| Sex, n (%) |  |  |  |  | < 0.001 |
| Male | 1442(48.36) | 457(53.64) | 426(52.34) | 559(41.34) |  |
| Female | 1542(51.64) | 388(46.36) | 415(47.66) | 739(58.66) |  |
| Race/ethnicity, n (%) |  |  |  |  | 0.02 |
| Non-Hispanic White People | 1707(57.17) | 460(70.46) | 464(73.11) | 783(79.55) |  |
| Non-Hispanic Black People | 666(22.37) | 227(17.91) | 203(14.69) | 236(9.82) |  |
| Mexican American People | 456(15.27) | 119(4.92) | 126(5.40) | 211(5.21) |  |
| Others | 155(5.19) | 39(6.72) | 48(6.80) | 68(5.42) |  |
| Smoking status, n (%) |  |  |  |  | < 0.0001 |
| Never | 1413(47.39) | 357(40.18) | 399(46.54) | 657(51.19) |  |
| Former | 1027(34.39) | 280(29.43) | 267(28.49) | 480(36.41) |  |
| Current | 544(18.22) | 208(30.39) | 175(24.97) | 161(12.40) |  |
| BMI (kg/m^2^), n (%) |  |  |  |  | 0.35 |
| <25.0 | 650(22.21) | 170(22.43) | 180(20.67) | 300(22.75) |  |
| 25.0–29.9 | 1032(35.15) | 284(30.38) | 304(37.64) | 444(34.61) |  |
| ≥30.0 | 1252(42.64) | 379(47.19) | 339(41.70) | 534(42.64) |  |
| Physical activity, n (%) |  |  |  |  | < 0.0001 |
| Sedentary | 923(30.91) | 310(30.08) | 278(24.87) | 335(18.90) |  |
| Insufficient | 1033(34.66) | 284(36.44) | 306(43.01) | 443(36.95) |  |
| Moderate | 444(14.87) | 114(14.88) | 113(12.54) | 217(17.79) |  |
| High | 584(19.56) | 137(18.61) | 144(19.58) | 303(26.36) |  |
| SBP (mmHg) | 136.89±0.62 | 136.83±1.07 | 137.04±1.08 | 136.84±0.84 | 0.99 |
| DBP (mmHg) | 73.59±0.46 | 75.08±0.96 | 72.92±0.88 | 73.10±0.62 | 0.18 |
| Diabetes, n (%) | 766(25.95) | 237(24.17) | 203(19.61) | 326(21.18) | 0.22 |
| Hyperlipidemia, n (%) | 2408(80.64) | 692(81.88) | 689(82.94) | 1027(79.62) | 0.3 |
| TG (mg/dL) | 162.61±4.40 | 170.65±7.95 | 155.57±5.23 | 162.24±9.18 | 0.31 |
| HDL (mg/dL) | 53.78±0.48 | 51.23±0.91 | 52.48±0.78 | 56.09±0.66 | < 0.001 |
| HbA1c (%) | 5.73±0.02 | 5.77±0.05 | 5.76±0.06 | 5.68±0.03 | 0.17 |
| Fast blood glucose (mg/dL) | 6.14±0.07 | 6.13±0.12 | 6.01±0.09 | 6.23±0.10 | 0.18 |
| Waist circumference (cm) | 103.24±0.42 | 104.91±0.87 | 103.10±0.70 | 102.33±0.65 | 0.07 |
| Energy intake (Kcal) | 2004.38±25.24 | 2053.40±50.60 | 1969.80±29.05 | 1995.64±36.69 | 0.34 |

Data are presented as weighted means ± SEs for continuous variables and unweighted numbers (weighted percentages) for categorical variables.

MDS-L, Low MIND score, the MIND diet score <7.5; MDS-M, Medium MIND score, the MIND diet score ≥8 and <8.5; MDS -H, High MIND score, the MIND diet score ≥8.5.

Abbreviations: MDS, the MIND diet scores; BMI, body mass index; SBP, systolic blood pressure; DBP, diastolic blood pressure; TG, triglyceride; HDL-C, high-density lipoprotein cholesterol; HbA1c, glycosylated hemoglobin, type A1C.

Table S5. Linear relationship between blood pressure and MDS among the entire population and hypertensive patients.

| Participant | Per one-score increase in MDS | | | |
| --- | --- | --- | --- | --- |
|  | β | *P* value | | |
| The entire participants |  |  | | |
| SBP (mmHg) | -0.41 | 0.033 | | |
| DBP (mmHg) | 0.08 | 0.639 | | |
| Participants with hypertension |  | |  |  |
| SBP (mmHg) | -0.45 | 0.285 | | |
| DBP (mmHg) | -0.08 | 0.804 | | |

The regression was adjusted for age, sex, race/ethnicity, smoking status, physical activity, BMI, diabetes, dyslipidemia, and energy intake.

Abbreviations: BMI, body mass index; MIND, the Mediterranean-DASH Diet Intervention for Neurodegenerative Delay diet score; MDS, the MIND diet score; SBP, systolic blood pressure; DBP, diastolic blood pressure.

Table S6. Logistic regression analysis for the prevalence of ASCVD and Cox regression analysis for the risk of all-cause and cardiovascular mortality according to the MIND diet score in the entire population.

| Model | Per one-score increases in MDS  OR/HR (95% CI) | OR/HR (95% CI) | | | *P* trend |
| --- | --- | --- | --- | --- | --- |
|  |  | MDS-L | MDS-M | MDS-H |  |
| ASCVD |  |  |  |  |  |
| Number of ASCVD/totals | 821/6887 | 256/1999 | 219/1847 | 346/3041 |  |
| Crude | 0.96 (0.89, 1.02) | 1.00 | 0.92 (0.69, 1.23) | 0.85 (0.66, 1.10) | 0.204 |
| Model 1 | 0.85 (0.78, 0.93) | 1.00 | 0.69 (0.49, 0.97) | 0.58 (0.42, 0.79) | 0.002 |
| Model 2 | 0.91 (0.83, 0.99) | 1.00 | 0.76 (0.54, 1.06) | 0.69 (0.51, 0.94) | 0.023 |
| Model 3 | 0.92 (0.84, 0.99) | 1.00 | 0.78 (0.55, 1.10) | 0.71 (0.52, 0.97) | 0.034 |
| All-cause mortality |  |  |  |  |  |
| Number of deaths/totals | 1087/6887 | 349/1999 | 310/1847 | 437/3041 |  |
| Crude | 0.92 (0.87, 0.97) | 1 | 1.03 (0.88, 1.20) | 0.812 (0.67, 0.98) | 0.024 |
| Model 1 | 0.82 (0.78, 0.86) | 1 | 0.78 (0.67, 0.91) | 0.554 (0.46, 0.66) | <0.001 |
| Model 2 | 0.87 (0.83, 0.92) | 1 | 0.88 (0.75, 1.03) | 0.68 (0.57, 0.81) | <0.001 |
| Model 3 | 0.87 (0.83, 0.92) | 1 | 0.88 (0.75, 1.03) | 0.68 (0.57, 0.81) | <0.001 |
| CV mortality |  |  |  |  |  |
| Number of deaths/totals | 377/6887 | 123/1999 | 108/1847 | 146/3041 |  |
| Crude | 0.88 (0.80, 0.95) | 1 | 0.91 (0.65, 1.28) | 0.65 (0.47, 0.90) | 0.007 |
| Model 1 | 0.78 (0.70, 0.86) | 1 | 0.70 (0.48, 1.03) | 0.45 (0.31, 0.64) | <0.001 |
| Model 2 | 0.84 (0.76, 0.92) | 1 | 0.80 (0.55, 1.15) | 0.56 (0.41, 0.78) | <0.001 |
| Model 3 | 0.85 (0.77, 0.93) | 1 | 0.819 (0.55, 1.18) | 0.58 (0.42, 0.79) | <0.001 |

Model 1: adjusted for age, sex, and race/ethnicity.

Model 2: further adjusted (from Model 1) for smoking status, BMI, and physical activity.

Model 3: further adjusted (from Model 2) for diabetes, dyslipidemia, and energy intake.

MDS-L, Low MIND score, the MIND diet score <7.5; MDS-M, Medium MIND score, the MIND diet score ≥8 and <8.5; MDS -H, High MIND score, the MIND diet score ≥8.5.

Abbreviations: ASCVD, atherosclerotic cardiovascular disease; BMI, body mass index; CV, cardiovascular; MDS, the MIND diet scores; ORs, odd ratio; HRs, hazard ratios; CIs, confidence intervals; MDS, the MIND diet score, the Mediterranean-DASH Diet Intervention for Neurodegenerative Delay diet score.

Table S7. Logistic regression analysis for the prevalence of ASCVD and Cox regression analysis for the risk of all-cause and cardiovascular mortality according to the MIND diet score among participants without hypertension.

| Model | Per one-score increases in MDS  OR/HR (95% CI) | OR/HR (95% CI) | | | *P* trend |
| --- | --- | --- | --- | --- | --- |
|  |  | MDS-L | MDS-M | MDS-H |  |
| ASCVD |  |  |  |  |  |
| Number of ASCVD/totals | 180/3903 | 53/1153 | 36/1006 | 91/1744 |  |
| Crude | 1.08 (0.92, 1.28) | 1.00 | 0.77 (0.45, 1.31) | 1.20 (0.69, 2.08) | 0.435 |
| Model 1 | 0.93 (0.76, 1.14) | 1.00 | 0.58 (0.34, 0.98) | 0.73 (0.42, 1.29) | 0.398 |
| Model 2 | 0.96 (0.77, 1.20) | 1.00 | 0.59 (0.33, 1.06) | 0.78 (0.42, 1.45) | 0.571 |
| Model 3 | 0.96 (0.77, 1.20) | 1.00 | 0.58 (0.32, 1.06) | 0.77 (0.42, 1.41) | 0.528 |
| All-cause mortality |  |  |  |  |  |
| Number of deaths/totals | 300/3903 | 95/1153 | 67/1006 | 138/1744 |  |
| Crude | 0.97 (0.88, 1.07) | 1.00 | 0.81 (0.51, 1.28) | 0.92 (0.64, 1.33) | 0.723 |
| Model 1 | 0.80 (0.72, 0.89) | 1.00 | 0.62 (0.39, 0.98) | 0.52 (0.36, 0.75) | 0.001 |
| Model 2 | 0.86 (0.77, 0.96) | 1.00 | 0.73 (0.46, 1.15) | 0.64 (0.44, 0.95) | 0.031 |
| Model 3 | 0.86 (0.77, 0.96) | 1.00 | 0.72 (0.45, 1.15) | 0.65 (0.44, 0.94) | 0.028 |
| CV mortality |  |  |  |  |  |
| Number of deaths/totals | 84/3903 | 53/1153 | 36/1006 | 91/1744 |  |
| Crude | 0.87 (0.72, 1.05) | 1.00 | 0.48 (0.20, 1.12) | 0.68 (0.37, 1.24) | 0.263 |
| Model 1 | 0.71 (0.55, 0.92) | 1.00 | 0.39 (0.16, 0.96) | 0.39 (0.18, 0.83) | 0.025 |
| Model 2 | 0.76 (0.58, 0.99) | 1.00 | 0.45 (0.19, 1.10) | 0.48 (0.21, 1.08) | 0.096 |
| Model 3 | 0.76 (0.58, 1.00) | 1.00 | 0.42 (0.17, 1.06) | 0.47 (0.21, 1.07) | 0.096 |

Model 1: adjusted for age, sex, and race/ethnicity.

Model 2: further adjusted (from Model 1) for smoking status, BMI, and physical activity.

Model 3: further adjusted (from Model 2) for diabetes, dyslipidemia, and energy intake.

MDS-L, Low MIND score, the MIND diet score <7.5; MDS-M, Medium MIND score, the MIND diet score ≥8 and <8.5; MDS -H, High MIND score, the MIND diet score ≥8.5.

Abbreviations: ASCVD, atherosclerotic cardiovascular disease; BMI, body mass index; MDS, the MIND diet scores; ORs, odd ratio; HRs, hazard ratios; CIs, confidence intervals; MDS, the MIND diet score, the Mediterranean-DASH Diet Intervention for Neurodegenerative Delay diet score; BMI, body mass index.

Table S8. Subgroup analyses of the associations between MIND diet score and hypertension among the entire participants.

| Subgroup | No. Hypertension/Total | OR (95% CI) | | | *P* for interaction |
| --- | --- | --- | --- | --- | --- |
|  |  | MDS-L | MDS-M | MDS-H |  |
| Age (years) |  |  |  |  | 0.918 |
| <60 | 1135/4284 | 1.00 | 0.87 (0.64, 1.18) | 0.71 (0.52, 0.95) |  |
| ≥60 | 1849/2603 | 1.00 | 1.10 (0.72, 1.70) | 0.87 (0.61, 1.22) |  |
| Sex |  |  |  |  | 0.492 |
| Male | 1443/3181 | 1.00 | 1.09 (0.79, 1.49) | 0.84 (0.62, 1.15) |  |
| Female | 1541/3706 | 1.00 | 0.78 (0.55, 1.10) | 0.69 (0.47, 0.99) |  |
| Race/ethnicity |  |  |  |  | 0.942 |
| White People | 1706/3078 | 1.00 | 0.93 (0.65, 1.32) | 0.79 (0.54, 1.14) |  |
| Non-White People | 1278/3809 | 1.00 | 0.96 (0.64, 1.44) | 0.71 (0.51, 0.99) |  |
| Smoking status |  |  |  |  | 0.263 |
| Never | 1415/3526 | 1.00 | 0.92 (0.66, 1.30) | 0.84 (0.57, 1.22) |  |
| Former/Current | 1569/3361 | 1.00 | 0.94 (0.67, 1.33) | 0.70 (0.50, 0.97) |  |
| BMI, kg/m^2^ |  |  |  |  | 0.108 |
| <30 | 1682/4438 | 1.00 | 1.11 (0.79, 1.56) | 0.79 (0.57, 1.08) |  |
| ≥30 | 1252/2341 | 1.00 | 0.73 (0.53, 1.02) | 0.75 (0.53, 1.06) |  |
| Physical activity |  |  |  |  | 0.121 |
| Sedentary/Insufficient | 1956/4395 | 1.00 | 1.04 (0.78, 1.40) | 0.71 (0.52, 0.98) |  |
| Moderate/High | 1028/2492 | 1.00 | 0.76 (0.55, 1.07) | 0.81 (0.56, 1.17) |  |
| Diabetes |  |  |  |  | 0.232 |
| Yes | 767/1021 | 1.00 | 0.62 (0.35, 1.10) | 0.65 (0.38, 1.10) |  |
| No | 2217/5866 | 1.00 | 0.98 (0.74, 1.29) | 0.77 (0.57, 1.03) |  |

All the models were adjusted for age, sex, race/ethnicity, smoking status, BMI, physical activity, diabetes, dyslipidemia, and energy intake.

MDS-L, Low MIND score, the MIND diet score <7.5; MDS-M, Medium MIND score, the MIND diet score ≥8 and <8.5; MDS -H, High MIND score, the MIND diet score ≥8.5.

Abbreviations: BMI, body mass index; MIND, the Mediterranean-DASH Diet Intervention for Neurodegenerative Delay diet score; MDS, the MIND diet score; MIND diet, Mediterranean-DASH Diet Intervention for Neurodegenerative Delay diet; ORs, odd ratio; CIs, confidence intervals.

Table S9. Subgroup analyses of the associations between MIND diet score and the prevalence of ASCVD among patients with hypertension

| Subgroup | No. ASCVD/Total | OR (95% CI) | | | *P* for interaction |
| --- | --- | --- | --- | --- | --- |
|  |  | MDS-L | MDS-M | MDS-H |  |
| Age (years) |  |  |  |  | 0.613 |
| <60 | 112/1135 | 1.00 | 0.80 (0.42, 1.53) | 0.49 (0.25, 0.97) |  |
| ≥60 | 529/1849 | 1.00 | 0.78 (0.50, 1.20) | 0.79 (0.55, 1.13) |  |
| Sex |  |  |  |  | 0.011 |
| Male | 351/1443 | 1.00 | 1.05 (0.54, 2.04) | 1.20 (0.82, 1.76) |  |
| Female | 290/1541 | 1.00 | 0.65 (0.38, 1.13) | 0.44 (0.26, 0.74) |  |
| Race/ethnicity |  |  |  |  | 0.173 |
| White People | 409/1706 | 1.00 | 0.86 (0.50, 1.50) | 0.83 (0.56, 1.25) |  |
| Non-White People | 232/1278 | 1.00 | 0.67 (0.37, 1.21) | 0.45 (0.26, 0.76) |  |
| Smoking status |  |  |  |  | 0.155 |
| Never | 242/1415 | 1.00 | 1.18 (0.59, 2.34) | 0.74 (0.37, 1.46) |  |
| Former/Current | 399/1569 | 1.00 | 0.62 (0.36, 1.06) | 0.68 (0.45, 1.03) |  |
| BMI, kg/m^2^ |  |  |  |  | 0.725 |
| <30 | 364/1682 | 1.00 | 0.75 (0.50, 1.14) | 0.66 (0.42, 1.02) |  |
| ≥30 | 257/1252 | 1.00 | 0.88 (0.49, 1.60) | 0.80 (0.49, 1.31) |  |
| Physical activity |  |  |  |  | 0.313 |
| Sedentary/Insufficient | 594/1956 | 1.00 | 0.75 (0.47, 1.21) | 0.57 (0.37, 0.86) |  |
| Moderate/High | 193/1028 | 1.00 | 0.81 (0.40, 1.62) | 1.03 (0.59, 1.81) |  |
| Diabetes |  |  |  |  | 0.670 |
| Yes | 225/767 | 1.00 | 0.62 (0.35, 1.12) | 0.68 (0.35, 1.33) |  |
| No | 416/2217 | 1.00 | 0.82 (0.48, 1.42) | 0.66 (0.47, 0.93) |  |

All the models were adjusted for age, sex, race/ethnicity, smoking status, BMI, physical activity, diabetes, dyslipidemia, and energy intake.

MDS-L, Low MIND score, the MIND diet score <7.5; MDS-M, Medium MIND score, the MIND diet score ≥8 and <8.5; MDS -H, High MIND score, the MIND diet score ≥8.5.

Abbreviations: ASCVD, atherosclerotic cardiovascular disease; BMI, body mass index; MIND, the Mediterranean-DASH Diet Intervention for Neurodegenerative Delay diet score; MDS, the MIND diet score; MIND diet, Mediterranean-DASH Diet Intervention for Neurodegenerative Delay diet; ORs, odd ratio; CIs, confidence intervals.

Table S10. Subgroup analyses of the associations between MIND diet score and all-cause mortality among patients with hypertension

| Subgroup | No. All-cause death/Total | HR (95% CI) | | | *P* for interaction |
| --- | --- | --- | --- | --- | --- |
|  |  | MDS-L | MDS-M | MDS-H |  |
| Age (years) |  |  |  |  | 0.043 |
| <60 | 95/1135 | 1.00 | 0.89 (0.54, 1.48) | 0.86 (0.48, 1.53) |  |
| ≥60 | 692/1849 | 1.00 | 0.91 (0.75, 1.11) | 0.67 (0.53, 0.84) |  |
| Sex |  |  |  |  | 0.037 |
| Male | 428/1443 | 1.00 | 1.03 (0.74, 1.23) | 0.87 (0.65, 1.17) |  |
| Female | 359/1541 | 1.00 | 0.83 (0.62, 1.12) | 0.53 (0.39, 0.74) |  |
| Race/ethnicity |  |  |  |  | 0.528 |
| White People | 529/1706 | 1.00 | 0.93 (0.76, 1.15) | 0.72 (0.59, 0.89) |  |
| Non-White People | 258/1278 | 1.00 | 0.79 (0.54, 1.16) | 0.48 (0.32, 0.72) |  |
| Smoking status |  |  |  |  | 0.205 |
| Never | 500/1682 | 1.00 | 0.83 (0.65, 1.05) | 0.63 (0.48, 0.82) |  |
| Former/Current | 262/1252 | 1.00 | 1.17 (0.79, 1.73) | 0.95 (0.68, 1.33) |  |
| BMI, kg/m^2^ |  |  |  |  | 0.708 |
| <30 | 5941956 | 1.00 | 0.84 (0.69, 1.03) | 0.66 (0.55, 0.80) |  |
| ≥30 | 193/1028 | 1.00 | 1.13 (0.73, 1.74) | 0.88 (0.60, 1.29) |  |
| Physical activity |  |  |  |  | 0.120 |
| Sedentary/Insufficient | 275/767 | 1.00 | 0.72 (0.48, 1.07) | 0.58 (0.45, 0.77) |  |
| Moderate/High | 512/2217 | 1.00 | 1.06 (0.85, 1.31) | 0.78 (0.62, 0.98) |  |
| Diabetes |  |  |  |  | 0.303 |
| Yes | 305/1415 | 1.00 | 1.10 (0.71, 1.70) | 0.79 (0.53, 1.78) |  |
| No | 482/1569 | 1.00 | 0.79 (0.64, 0.96) | 0.66 (0.54, 0.80) |  |

All the models were adjusted for age, sex, race/ethnicity, smoking status, BMI, physical activity, diabetes, dyslipidemia, and energy intake.

MDS-L, Low MIND score, the MIND diet score <7.5; MDS-M, Medium MIND score, the MIND diet score ≥8 and <8.5; MDS -H, High MIND score, the MIND diet score ≥8.5.

Abbreviations: BMI, body mass index; MIND, the Mediterranean-DASH Diet Intervention for Neurodegenerative Delay diet score; MDS, the MIND diet score; MIND diet, Mediterranean-DASH Diet Intervention for Neurodegenerative Delay diet; HRs, hazard ratios; CIs, confidence intervals.

Table S11. Subgroup analyses of the associations between MIND diet score and CV mortality among patients with hypertension

| Subgroup | No. CV death/Total | HR (95% CI) | | | *P* for interaction |
| --- | --- | --- | --- | --- | --- |
|  |  | MDS-L | MDS-M | MDS-H |  |
| Age (years) |  |  |  |  | 0.588 |
| <60 | 261/1135 | 1.00 | 0.97 (0.60, 1.57) | 0.74 (0.44, 1.22) |  |
| ≥60 | 32/1849 | 1.00 | 0.91 (0.35, 2.34) | 0.39 (0.15, 0.99) |  |
| Sex |  |  |  |  | 0.264 |
| Male | 161/1443 | 1.00 | 1.19 (0.75, 1.89) | 0.82 (0.57, 1.19) |  |
| Female | 132/1541 | 1.00 | 0.82 (0.43, 1.55) | 0.49 (0.26, 0.93) |  |
| Race/ethnicity |  |  |  |  | 0.016 |
| White People | 206/1706 | 1.00 | 1.31 (0.81, 2.10) | 0.74 (0.47, 1.16) |  |
| Non-White People | 87/1278 | 1.00 | 0.40 (0.16, 0.96) | 0.32 (0.14, 0.71) |  |
| Smoking status |  |  |  |  | 0.719 |
| Never | 118/1415 | 1.00 | 1.00 (0.45, 2.26) | 0.53 (0.20, 1.19) |  |
| Former/Current | 175/1569 | 1.00 | 0.83 (0.54, 1.28) | 0.65 (0.46, 0.90) |  |
| BMI, kg/m^2^ |  |  |  |  | 0.958 |
| <30 | 182/1682 | 1.00 | 0.88 (0.55, 1.42) | 0.62(0.38, 1.01) |  |
| ≥30 | 105/1252 | 1.00 | 0.95 (0.50, 1.81) | 0.57 (0.30, 1.08) |  |
| Physical activity |  |  |  |  | 0.151 |
| Sedentary/Insufficient | 229/1956 | 1.00 | 0.74 (0.46, 1.20) | 0.53 (0.35, 0.82) |  |
| Moderate/High | 64/1028 | 1.00 | 1.19 (0.83, 5.77) | 0.93 (0.38, 2.29) |  |
| Diabetes |  |  |  |  | 0.170 |
| Yes | 111/767 | 1.00 | 0.79 (0.44, 1.40) | 0.39 (0.21, 0.74) |  |
| No | 182/2217 | 1.00 | 1.13 (0.62, 2.05) | 0.82 (0.54, 1.23) |  |

All the models were adjusted for age, sex, race/ethnicity, smoking status, BMI, physical activity, diabetes, dyslipidemia, and energy intake.

MDS-L, Low MIND score, the MIND diet score <7.5; MDS-M, Medium MIND score, the MIND diet score ≥8 and <8.5; MDS -H, High MIND score, the MIND diet score ≥8.5.

Abbreviations: BMI, body mass index; CV, cardiovascular; MIND, the Mediterranean-DASH Diet Intervention for Neurodegenerative Delay diet score; MDS, the MIND diet score; MIND diet, Mediterranean-DASH Diet Intervention for Neurodegenerative Delay diet; HRs, hazard ratios; CIs, confidence intervals.

Table S12. Sensitivity Analyses of the associations between MIND diet score and all-cause/CV mortality among hypertensive patients

| Analysis | HR (95% CI) | | | *P* trend |
| --- | --- | --- | --- | --- |
|  | MDS-L | MDS-M | MDS-H |  |
| Excluding Hispanic participants (N=609) |  |  |  |  |
| All-cause mortality | 1.00 | 0.93 (0.75, 1.12) | 0.81 (0.65, 1.00) | 0.048 |
| CV mortality | 1.00 | 0.91 (0.61, 1.37) | 0.63 (0.41, 0.95) | 0.023 |
| Excluding Mexican American and other participants (N=2375) |  |  |  |  |
| All-cause mortality | 1.00 | 0.96 (0.80, 1.14) | 0.78 (0.64, 0.96) | 0.014 |
| CV mortality | 1.00 | 0.93 (0.63, 1.38) | 0.66 (0.46, 0.96) | 0.023 |
| Excluding participants who died within 1 year of follow-up (N=2977) | |  |  |  |
| All-cause mortality | 1.00 | 0.93 (0.79, 1.09) | 0.68 (0.57,0.81) | <0.001 |
| CV mortality | 1.00 | 0.89 (0.65, 1.22) | 0.60 (0.42, 0.85) | 0.003 |
| Excluding participants with cerebral diseases (N=2749) |  |  |  |  |
| All-cause mortality | 1.00 | 0.90 (0.75, 1.09) | 0.68 (0.57, 0.81) | <0.001 |
| CV mortality | 1.00 | 0.85 (0.59, 1.21) | 0.63 (0.43, 0.92) | 0.003 |

All the models were adjusted for age, sex, race/ethnicity, smoking status, BMI, physical activity, diabetes, dyslipidemia, and energy intake.

MDS-L, Low MIND score, the MIND diet score <7.5; MDS-M, Medium MIND score, the MIND diet score ≥8 and <8.5; MDS -H, High MIND score, the MIND diet score ≥8.5.

Abbreviations: BMI, body mass index; CV, cardiovascular; MIND, the Mediterranean-DASH Diet Intervention for Neurodegenerative Delay diet score; MDS, the MIND diet score; HRs, hazard ratios; CIs, confidence intervals; MIND diet, Mediterranean-DASH Diet Intervention for Neurodegenerative Delay diet; HRs, hazard ratios; CIs, confidence intervals.

Table S13. Logistic regression analysis for the prevalence of hypertension and ASCVD and Cox regression analysis for the risk of all-cause and cardiovascular mortality according to the mean MIND diet score of each MDS category.

|  | The mean MDS | | | OR/HR (95% CI)^1^ | *P* for trend |
| --- | --- | --- | --- | --- | --- |
|  | MDS-L | MDS-M | MDS-H |  |  |
| HTN^2^ | 6.45 | 7.75 | 9.45 | 0.91(0.84, 0.99) | 0.045 |
| ASCVD^3^ | 6.45 | 7.75 | 9.45 | 0.90(0.83, 0.99) | 0.046 |
| All-cause mortality^3^ | 6.52 | 7.75 | 9.46 | 0.88(0.83, 0.93) | <0.001 |
| CV mortality^3^ | 6.52 | 7.75 | 9.46 | 0.84(0.76, 0.93) | 0.010 |

1: adjusted for age, sex, race/ethnicity, smoking status, BMI, physical activity, diabetes, dyslipidemia, and energy intake.

2: in the entire participants;

3: in the participants with hypertension;

MDS-L, Low MIND score, the MIND diet score <7.5; MDS-M, Medium MIND score, the MIND diet score ≥8 and <8.5; MDS -H, High MIND score, the MIND diet score ≥8.5.

Abbreviations: ASCVD, atherosclerotic cardiovascular disease; BMI, body mass index; MDS, the MIND diet scores; ORs, odd ratio; HRs, hazard ratios; CIs, confidence intervals; MDS, the MIND diet score, the Mediterranean-DASH Diet Intervention for Neurodegenerative Delay diet score; BMI, body mass index.

Table S14. Associations of individual food components of the MIND diet with the prevalence of hypertension, the prevalence of ASCVD, the risk of all-cause and cardiovascular mortality.

| Components of MIND diet | The mean points (hypertensive patients/whole population) | Adjusted OR/HR (95% CI)^1^ | | | |
| --- | --- | --- | --- | --- | --- |
|  |  | HTN^2^ | ASCVD^3^ | All-cause Mortality^3^ | CV Mortality^3^ |
| Green leafy vegetables | 0.16/0.16 | 0.98(0.64, 1.49) | 0.68(0.40, 1.14) | **0.58 (0.43, 0.79)** | **0.49(0.32, 0.75)** |
| Other vegetables | 0.12/0.12 | 1.02(0.65, 1.62) | 1.35(0.79, 2.30) | 0.86 (0.56, 1.31) | 0.96(0.67, 1.38) |
| Berries | 0.24/0.24 | 0.87(0.70, 1.08) | 0.95(0.69, 1.32) | 0.90 (0.74, 1.09) | 0.77(0.54, 1.10) |
| Nuts | 0.32/0.32 | 0.90(0.69, 1.17) | 0.77(0.56, 1.05) | **0.66 (0.54, 0.81)** | **0.69(0.49, 0.96)** |
| Butter, Margarine | 0.99/0.99 | 0.49(0.20, 1.21) | 1.95(0.64, 5.99) | 0.78 (0.40, 1.53) | 0.66(0.22, 2.00) |
| Cheese | 0.71/0.69 | 1.22(0.96, 1.54) | 1.46(0.86, 2.48) | 0.80 (0.57, 1.13) | 0. 80(0.52, 1.22) |
| Whole Grains | 0.63/0.61 | 0.92(0.75, 1.12) | 0.97(0.74, 1.27) | 0.84 (0.70, 1.02) | 0.85(0.64, 1.13) |
| Fish (not fried) | 0.35/0.36 | 0.86(0.68, 1.08) | 0.92(0.58, 1.46) | 0.82 (0.66, 1.01) | **0.56(0.44, 0.72)** |
| Beans | 0.33/0.33 | 0.87(0.68, 1.10) | 0.97(0.66, 1.41) | 0.92 (0.76, 1.11) | 0.96(0.69, 1.33) |
| Poultry (not fried) | 0.38/0.41 | 0.91(0.72, 1.15) | **0.75(0.59, 0.95)** | 0.94 (0.76, 1.16) | 0.88(0.59, 1.31) |
| Red meat and products | 0.98/0.98 | 0.88(0.31, 2.50) | **0.30(0.11, 0.81)** | **0.24 (0.10, 0.57)** | **0.85(0.77, 0.93)** |
| Fast fried foods | 0.81/0.79 | 0.93(0.65, 1.33) | 0.78(0.46, 1.35) | 1.04(0.73, 1.47) | 1.12(0.62, 2.02) |
| Pastries & Sweets | 0.91/0.92 | 1.04(0.59, 1.81) | 1.15(0.67, 1.98) | 0.98(0.60, 1.58) | 1.48(0.62, 3.54) |
| Wine | 0.19/0.21 | 0.76(0.52 1.11) | **0.36(0.24, 0.52)** | **0.50(0.35, 0.72)** | **0.51(0.30, 0.87)** |

1: adjusted for age, sex, race/ethnicity, smoking status, BMI, physical activity, diabetes, dyslipidemia, and energy intake.

2: in the entire participants;

3: in the participants with hypertension;

Abbreviations: ASCVD, atherosclerotic cardiovascular disease; BMI, body mass index; MDS, the MIND diet scores; ORs, odd ratio; HRs, hazard ratios; CIs, confidence intervals; MDS, the MIND diet score, the Mediterranean-DASH Diet Intervention for Neurodegenerative Delay diet score; BMI, body mass index.


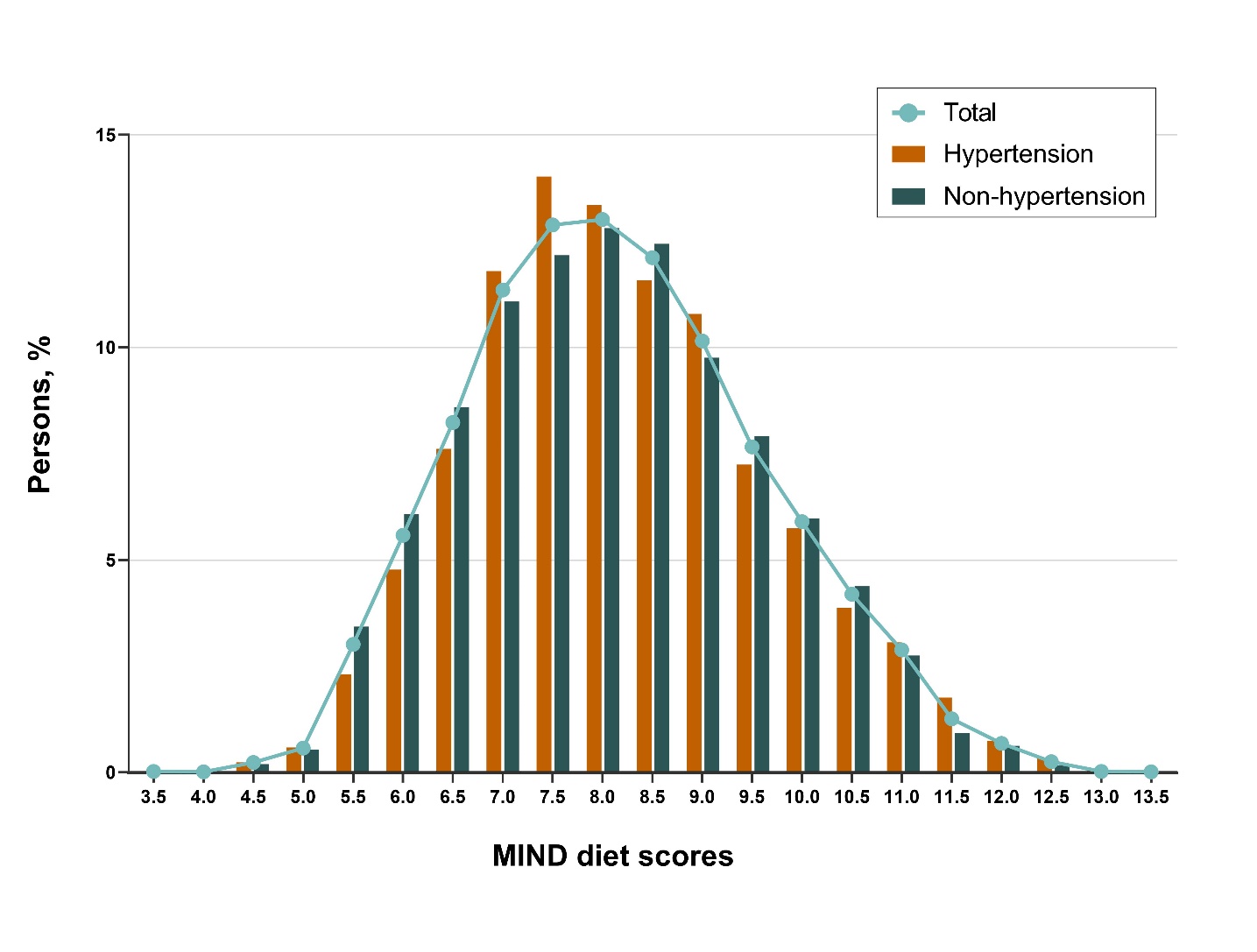
.

Figure S1. The MIND diet scores of included participants grouped by hypertension.

Abbreviations: MIND diet, Mediterranean-DASH Diet Intervention for Neurodegenerative Delay diet; MDS, the MIND diet scores


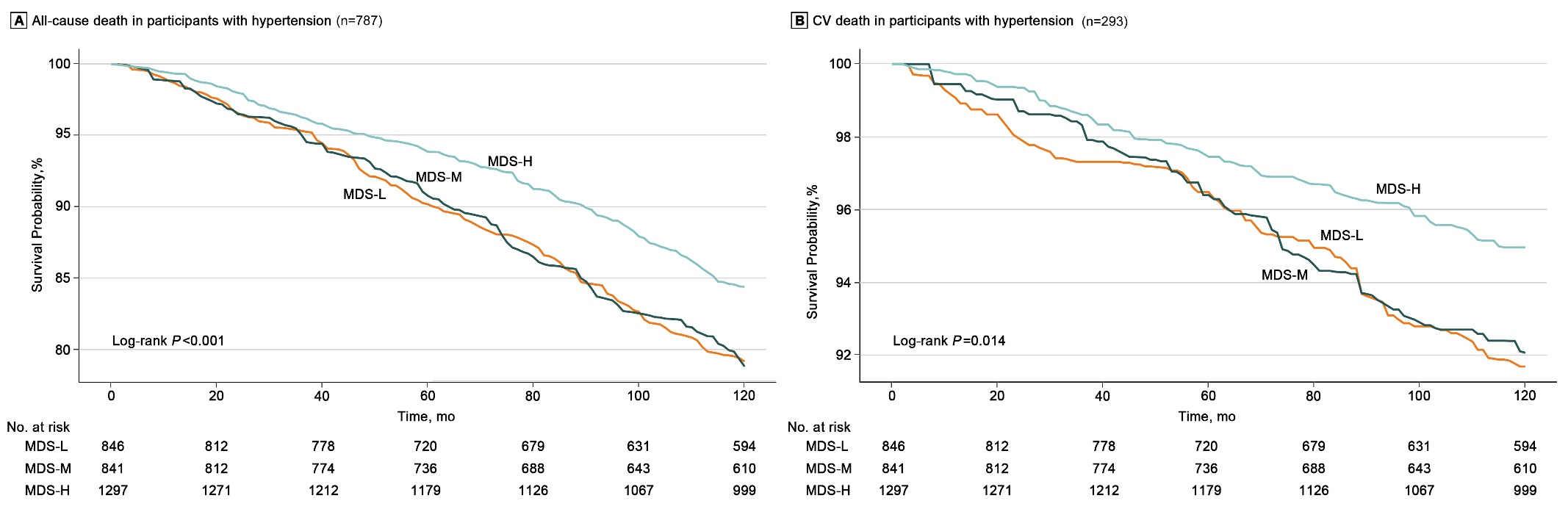


Figure S2. Kaplan-Meier curves for the all-cause and CV mortality in groups of MDS-L, MDS-M, and MDS-H in hypertensive patients.

MDS-L, Low MIND score, the MIND diet score <7.5; MDS-M, Medium MIND score, the MIND diet score ≥8 and <8.5; MDS -H, High MIND score, the MIND diet score ≥8.5.

Abbreviations: CV, cardiovascular; MIND diet, Mediterranean-DASH Diet Intervention for Neurodegenerative Delay diet; MDS, the MIND diet scores.


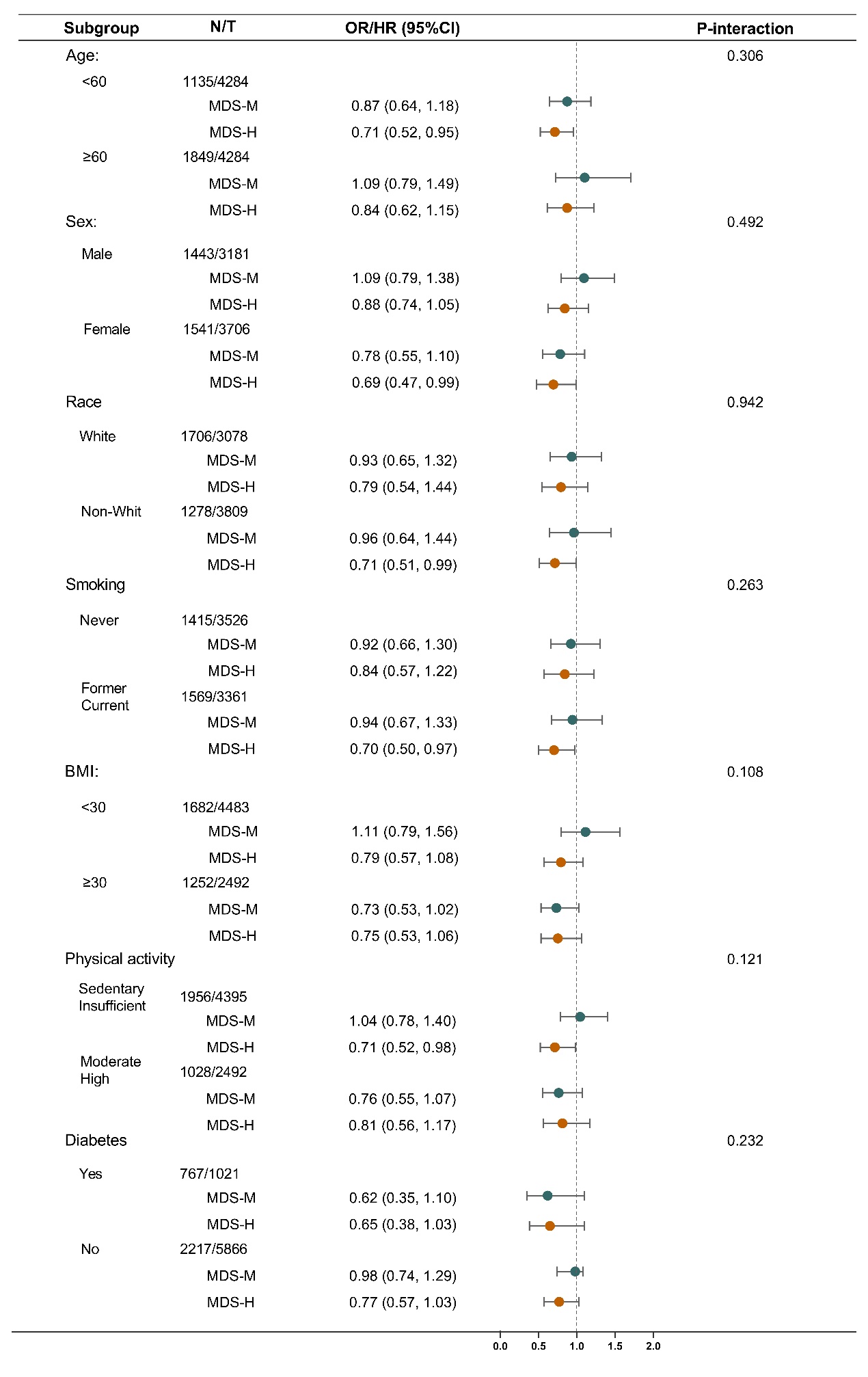
 Figure S3. Subgroup analysis for the prevalence of hypertension in the entire population.

MDS-M, Medium MIND score, the MIND diet score ≥8 and <8.5; MDS -H, High MIND score, the MIND diet score ≥8.5.

Abbreviations: BMI, body mass index; MIND diet, Mediterranean-DASH Diet Intervention for Neurodegenerative Delay diet; MDS, the MIND diet scores.


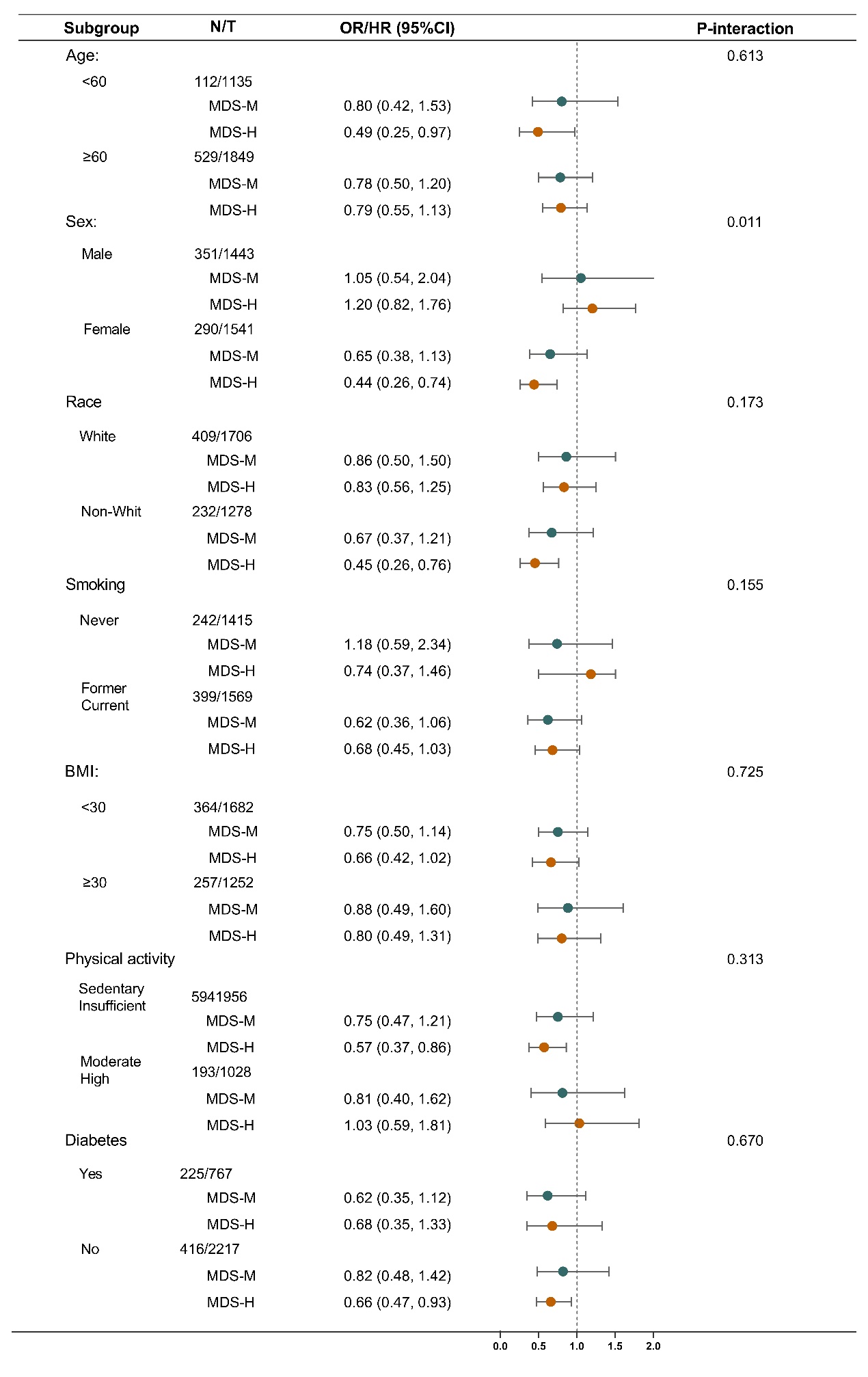
 Figure S4. Subgroup analysis for the prevalence of ASCVD in hypertensive patients.

MDS-M, Medium MIND score, the MIND diet score ≥8 and <8.5; MDS -H, High MIND score, the MIND diet score ≥8.5.

Abbreviations: ASCVD, atheroclerotic cardiovascular disease; BMI, body mass index; MIND diet, Mediterranean-DASH Diet Intervention for Neurodegenerative Delay diet; MDS, the MIND diet scores.


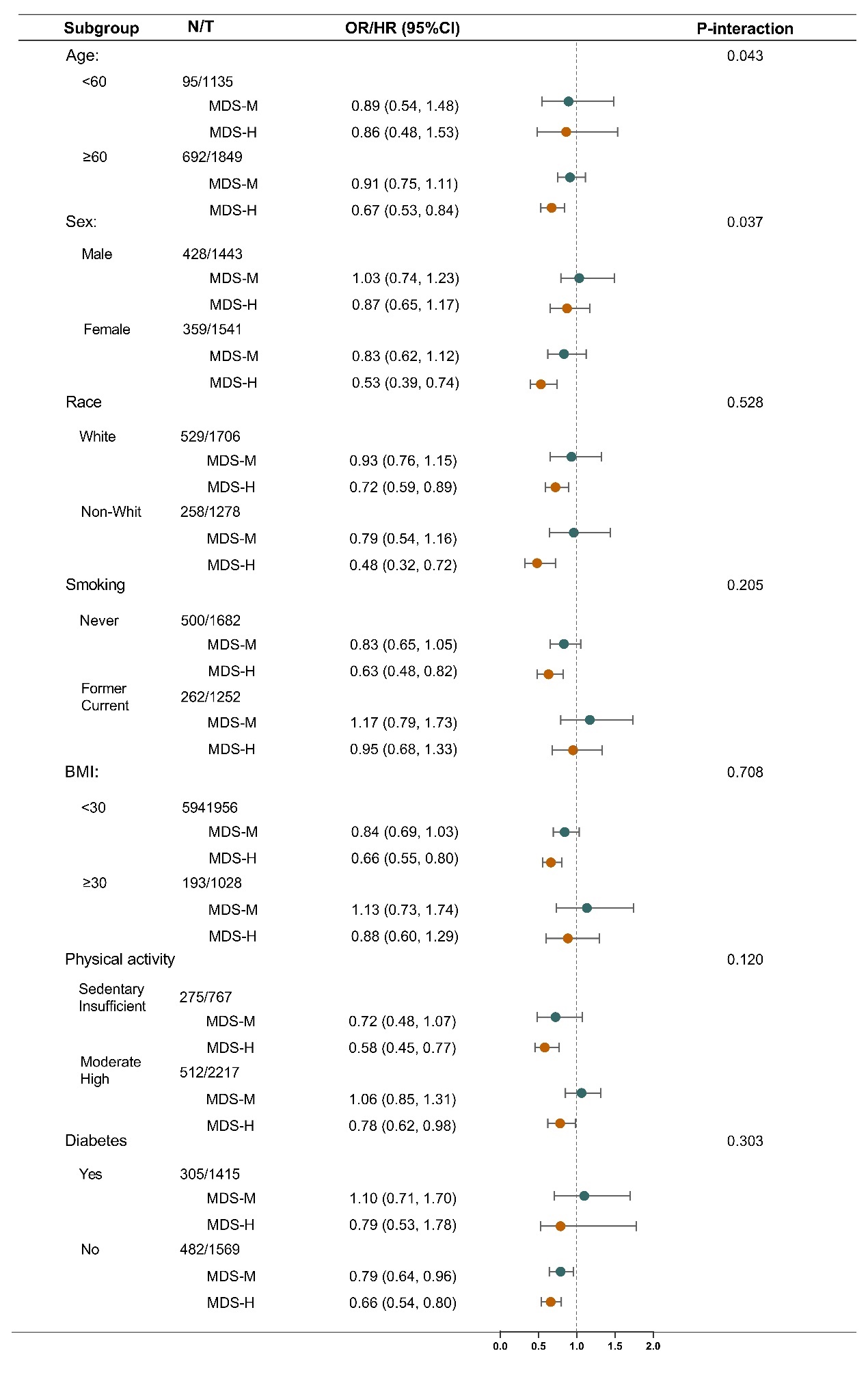
 Figure S5. Subgroup analysis for the risk of all-cause death in hypertensive patients.

MDS-M, Medium MIND score, the MIND diet score ≥8 and <8.5; MDS -H, High MIND score, the MIND diet score ≥8.5.

Abbreviations: BMI, body mass index; MIND diet, Mediterranean-DASH Diet Intervention for Neurodegenerative Delay diet; MDS, the MIND diet scores.


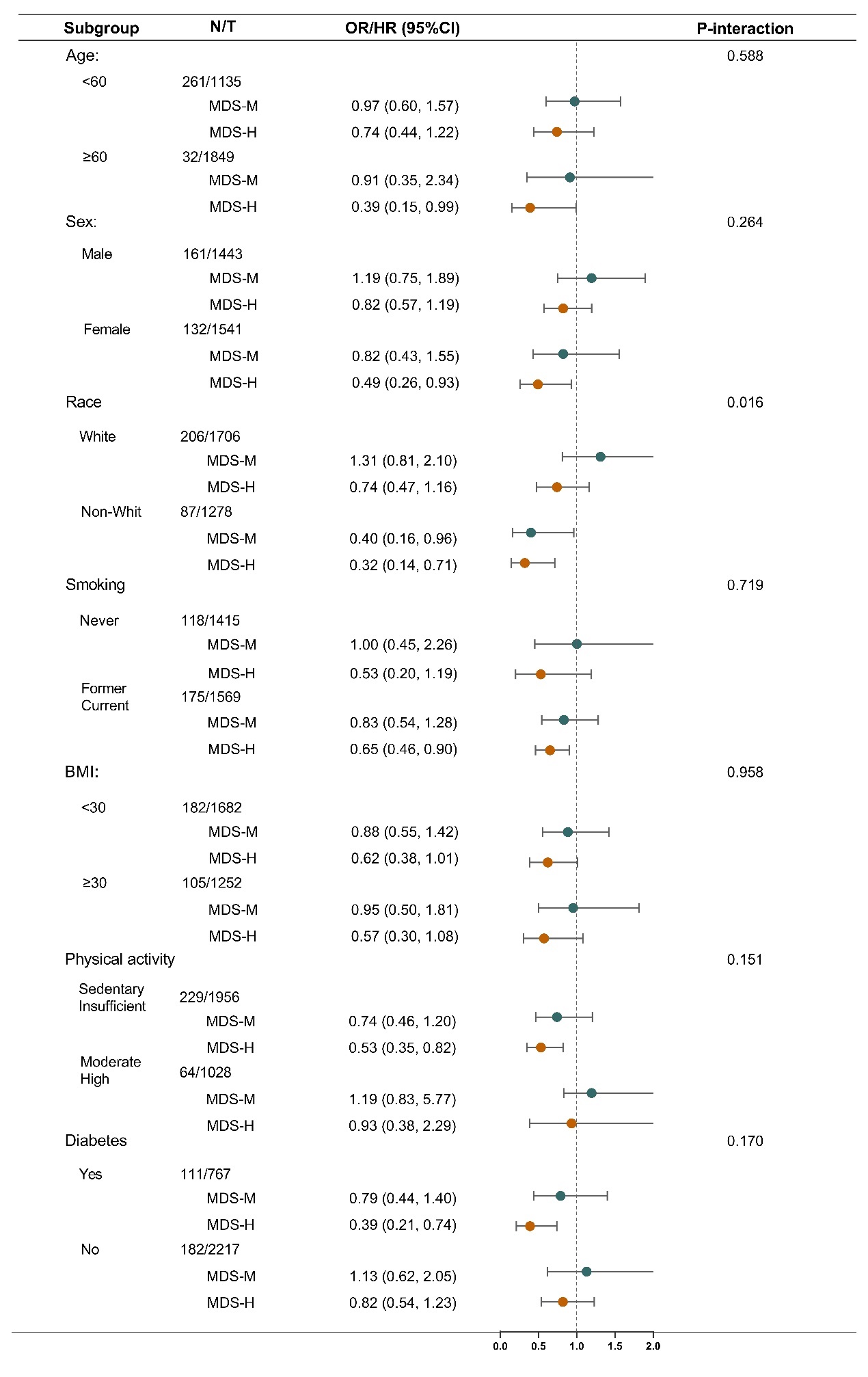
 Figure S6. Subgroup analysis for the risk of CV death in hypertensive patients.

MDS-M, Medium MIND score, the MIND diet score ≥8 and <8.5; MDS -H, High MIND score, the MIND diet score ≥8.5.

Abbreviations: BMI, body mass index; MIND diet, Mediterranean-DASH Diet Intervention for Neurodegenerative Delay diet; MDS, the MIND diet scores.

**Supplementary References**

1. CDC. NHANES Analytic Guidelines 2005. Available from: http://www.cdc.gov/nchs/nhanes/survey_methods.htm
2. CDC. NHANES Home Page. Available from: http://www.cdc.gov/nchs/about/major/nhanes/nhanes2003-2004/questexam03_04.htm
